# Pyruvate Kinase M (PKM) binds ribosomes in a poly-ADP ribosylation dependent manner to induce translational stalling

**DOI:** 10.1093/nar/gkad440

**Published:** 2023-05-24

**Authors:** Nevraj S Kejiou, Lena Ilan, Stefan Aigner, Enching Luo, Tori Tonn, Hakan Ozadam, Muyoung Lee, Gregory B Cole, Ines Rabano, Nishani Rajakulendran, Brian A Yee, Hamed S Najafabadi, Trevor F Moraes, Stephane Angers, Gene W Yeo, Can Cenik, Alexander F Palazzo

**Affiliations:** Department of Biochemistry, University of Toronto, Toronto, ON, Canada; Department of Biochemistry, University of Toronto, Toronto, ON, Canada; Department of Cellular and Molecular Medicine, University of California San Diego, La Jolla, CA, USA; Department of Cellular and Molecular Medicine, University of California San Diego, La Jolla, CA, USA; Department of Molecular Biosciences, University of Texas at Austin, Austin, TX, USA; Department of Molecular Biosciences, University of Texas at Austin, Austin, TX, USA; Department of Molecular Biosciences, University of Texas at Austin, Austin, TX, USA; Department of Biochemistry, University of Toronto, Toronto, ON, Canada; Department of Cellular and Molecular Medicine, University of California San Diego, La Jolla, CA, USA; Leslie Dan Faculty of Pharmacy, University of Toronto, Toronto, ON, Canada; Department of Cellular and Molecular Medicine, University of California San Diego, La Jolla, CA, USA; Department of Human Genetics, McGill University, Montreal, QC, Canada; McGill University and Genome Quebec Innovation Centre, Montreal, QC, Canada; Department of Biochemistry, University of Toronto, Toronto, ON, Canada; Department of Biochemistry, University of Toronto, Toronto, ON, Canada; Leslie Dan Faculty of Pharmacy, University of Toronto, Toronto, ON, Canada; Department of Cellular and Molecular Medicine, University of California San Diego, La Jolla, CA, USA; Department of Molecular Biosciences, University of Texas at Austin, Austin, TX, USA; Department of Biochemistry, University of Toronto, Toronto, ON, Canada

## Abstract

In light of the numerous studies identifying post-transcriptional regulators on the surface of the endoplasmic reticulum (ER), we asked whether there are factors that regulate compartment specific mRNA translation in human cells. Using a proteomic survey of spatially regulated polysome interacting proteins, we identified the glycolytic enzyme Pyruvate Kinase M (PKM) as a cytosolic (i.e. ER-excluded) polysome interactor and investigated how it influences mRNA translation. We discovered that the PKM-polysome interaction is directly regulated by ADP levels–providing a link between carbohydrate metabolism and mRNA translation. By performing enhanced crosslinking immunoprecipitation-sequencing (eCLIP-seq), we found that PKM crosslinks to mRNA sequences that are immediately downstream of regions that encode lysine- and glutamate-enriched tracts. Using ribosome footprint protection sequencing, we found that PKM binding to ribosomes causes translational stalling near lysine and glutamate encoding sequences. Lastly, we observed that PKM recruitment to polysomes is dependent on poly-ADP ribosylation activity (PARylation)—and may depend on co-translational PARylation of lysine and glutamate residues of nascent polypeptide chains. Overall, our study uncovers a novel role for PKM in post-transcriptional gene regulation, linking cellular metabolism and mRNA translation.

## Graphical Abstract

**Graphical Abstract:**
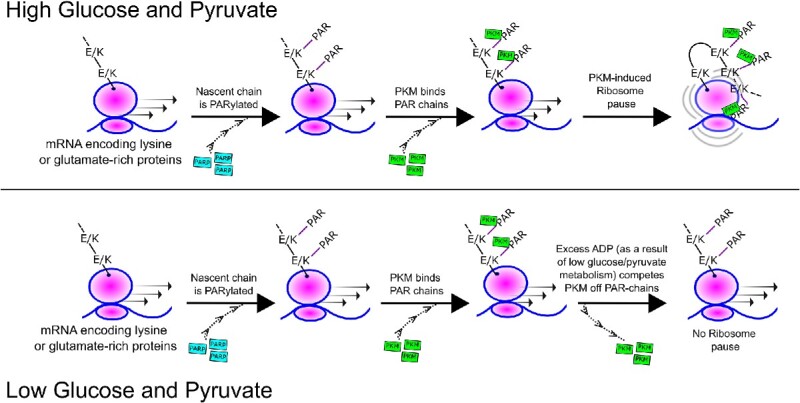

## INTRODUCTION

The regulation of mRNA stability, translation and localization is critical for cellular function. Post-transcriptional regulators of mRNAs include RNA-binding proteins (RBPs), ribosome interacting proteins, and nascent-chain associated factors. Various groups have identified post-transcriptional regulators by assaying for mRNA or ribosome interactions. Of particular interest are putative post-transcriptional regulators lacking annotated RNA-binding domains, known as ‘noncanonical’, ‘unconventional’ or ‘enigm-’ RBPs, and tend to include many metabolic enzymes hinting at a potential connection between metabolism and mRNA regulation (1–16). In some cases, these putative regulators have been documented to interact with the mRNA, in other cases the ribosomes, and rarely with the nascent chain. Most of these studies have been conducted in model systems, such as yeast, flies, and mammalian cell lines, but recently these have been extended to more physiological contexts such as in mouse organs (17).

Additionally, an overlooked aspect of mRNA biology is the spatial organization of post-transcriptional regulators within cells. This spatial organization contributes to the local regulation of mRNA translation in response to subcellular demands (18) and compartment-specific stress such as the unfolded protein response in the lumen of the endoplasmic reticulum (ER) (19). Although there are known differences between the regulation of mRNA translation on the surface of the ER versus the cytosol (20–25), most studies of spatially restricted post-transcriptional regulators have focused on ER-bound ribosomes (26–28). However, to understand how mRNAs are spatially regulated, we must also understand how these factors are enriched in free cytosolic (i.e. ER-excluded) polysomes.

To address how cytosolic and ER-associated polysomes are differentially regulated, we combined cellular fractionation and high-speed centrifugation to isolate both cytosolic and ER ribosomes and identified their proteomic composition by mass spectrometry. We focused our efforts on Pyruvate Kinase M (PKM), as our preliminary results suggested that its association to ribosomes was restricted to cytosolic polysomes and sensitive to glucose/pyruvate-starvation. Canonically, PKM produces pyruvate from phosphoenolpyruvate, while generating ATP from substrate-level phosphorylation. PKM has been also implicated in the Warburg effect, where it may shunt glycolytic substrates towards anabolic processes rather than oxidative phosphorylation (29–31). Beyond metabolism, PKM has been reported to act as a protein kinase to regulate various processes such as cell proliferation, DNA repair, mitotic progression, and transcription (32–36). PKM lacks any identifiable RNA-binding domain, making it an ideal model for understanding how these non-canonical RNA-binding proteins are interacting with RNA. Previously, it had been found that PKM bound directly to ribosomes, likely near the A site, and altered mRNA translation (10), although the details and nature of this regulation were unclear. We found that PKM crosslinked with the open reading frames (ORFs) of mRNAs whose protein products are either cytosolic or nucleoplasmic. These interactions occurred just downstream of regions encoding glutamate or lysine. Furthermore, we demonstrated that PKM promotes ribosome stalling in the vicinity of glutamate- and lysine-encoding regions. Lastly, we found that PKM recruitment to ribosomes is dependent on poly-ADP ribosylation activity and may rely on co-translational PARylation of nascent polypeptides–a completely novel co-translational modification. Our data suggests that this interaction is disrupted by increases in cellular ADP, thus linking the cellular metabolic state to the regulation of mRNA translation.

## MATERIALS AND METHODS

### Cells, growth conditions, and lentiviral mediated depletion of PKM

U2OS, HepG2, HEK293T and HEK293F cells were maintained in DMEM supplemented with 10% fetal bovine serum (FBS) and 1% penicillin/streptomycin (P/S) at 37°C and 5% CO_2_. Short term glucose starvation was carried out via incubation with glucose- and pyruvate-free DMEM (Gibco Cat#11966–025) supplemented with 10% FBS and 1% P/S with either 20 mM 2-deoxyglucose (D8375 Sigma) or vehicle (dH_2_O) for 3 h. For poly-ADP ribosylation inhibition, U2OS cells were treated with 15 μM Olaparib or DMSO vehicle for 25 min. For mitotic arrest experiments, approximately 8.8 × 10^6^ U2OS cells were synchronized toward G1/S by growing in medium supplemented with 2 mM thymidine for 16 h, followed by 24 μM deoxycytidine for 8 h, followed by 2 mM thymidine for 16 h, and lastly by 24 μM deoxycytidine for 2 h. 100 ng/ml of nocodazole was added for 18 h to mitotically arrest synchronized cells. Mitotic cells were collected by vigorously washing tissue culture dish with PBS.

For lentiviral depletion of PKM, lentiviruses were generated as described previously (26), using clone TRCN0000199494 (Sigma; CCGGGCCCGA GGCTTCTTCA AGAAGCTCGA GCTTCTTGAA GAAGCCTCGG GCTTTTTTG). Infected cells were pre-treated with 8 μg/ml hexadimethrine bromide. Cells were selected with 2 μg/ml puromycin media for 6 days.

### Cell fractionation and oligo-dT affinity chromatography

To isolate crude polysomal fractions, 75 × 10^6^ U2OS cells were treated with growth medium supplemented with 10 μg/ml cycloheximide for 30 min. Cells were collected by trypsinization, then sedimented at 800 *g* for 2 min, washed twice with ice cold PBS containing 10 μg/ml cycloheximide, washed once with ice cold Phy Buffer (150 mM Potassium Acetate, 5 mM Magnesium Acetate, 20 mM HEPES–KOH pH 7.4, 5 mM DTT, protease inhibitor cocktail [Roche], 10 μg/ml cycloheximide). Cell pellets were then resuspended in 1 ml Phy Buffer. Cells were extracted by adding an equal volume of cold Phy Buffer + 0.08% digitonin and gently inverting the tube to allow the detergent to mix. The solution was then centrifuged at 800 *g* for 2 min to produce a suspension (cytosolic fraction, C1) and pellet (P1). The pellet was resuspended in additional 1 ml of cold Phy Buffer and extracted with an equal volume of cold Phy Buffer and 0.08% digitonin. The solution was then centrifuged at 800 *g* for 2 min to produce a suspension (C2) and pellet (ER + nuclear fraction, P2). The pellet was then resuspended in 1 ml cold Phy Buffer and extracted by adding an equal volume (1 ml) of Phy Buffer + 0.05% TritonX-100. This sample was then centrifuged at 800 *g* for 2 min to produce a suspension (ER fraction) and pellet (nuclear fraction). Cytosolic (C1) and ER fractions were then centrifuged at 10 000 *g* for 10 min to remove contaminating organelles such as mitochondria and nuclei. These fractions were analyzed for protein or further fractionated.

**Figure 1.**
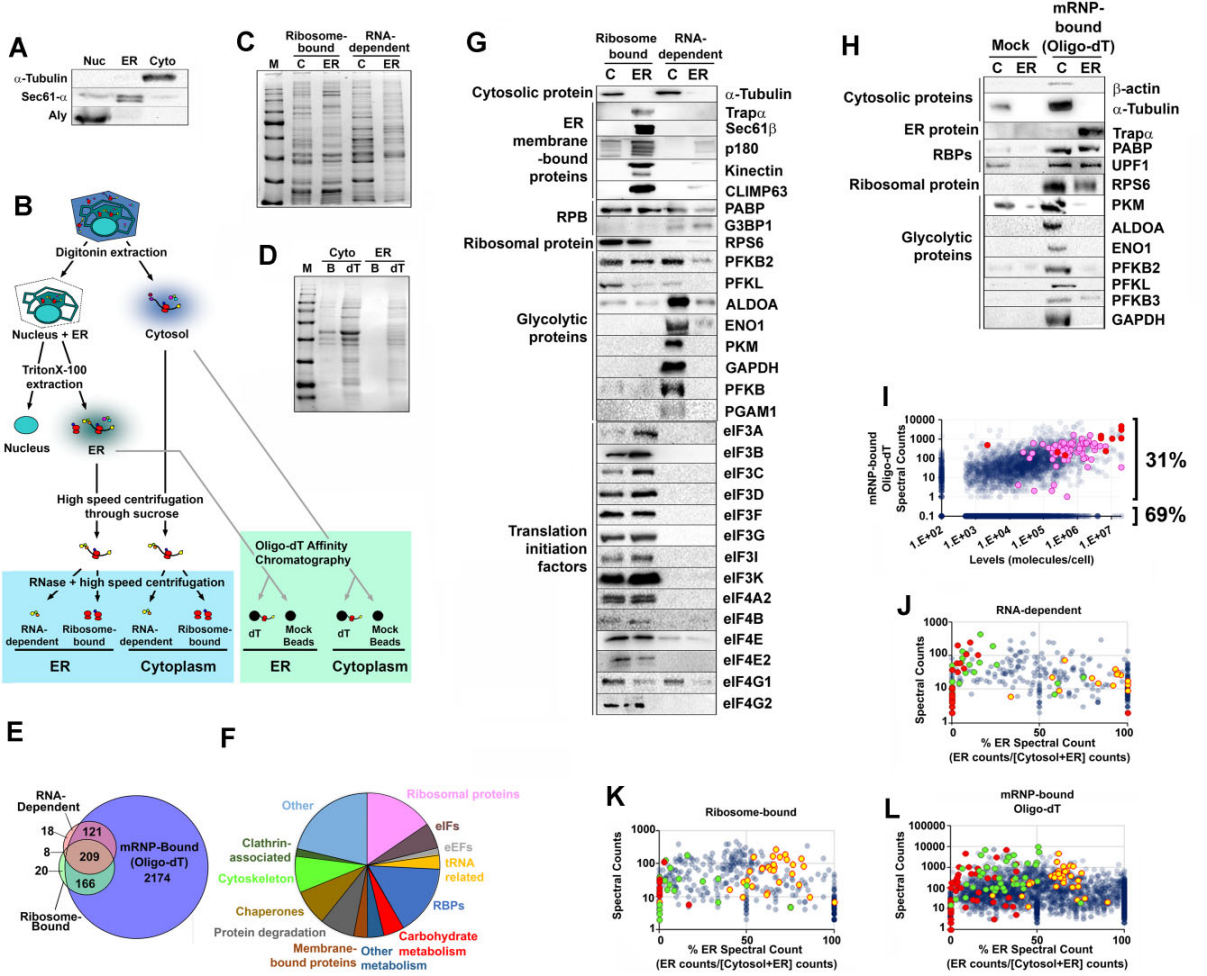
The distribution of different classes of proteins between cytosolic and ER polysomes. (**A**) U2OS cell fractions probed for a cytosolic marker (α-tubulin), an ER-marker (Sec61α) and a nuclear marker (Aly). (**B**) Schematic representation of the cell fractionation protocol. Crude polysome fractions are in the blue box, oligo-dT affinity chromatography fractions are in the green box. (**C**, **D**) Coomassie stains of the various fractions (‘M’: molecular weight marker, ‘C’: cytosol, ‘B’: mock beads, ‘dT’: oligo-dT). (**E**) The set of proteins enriched in either the two crude polysome fractions (RNA-dependent: red, or Ribosome-bound: green) or the oligo-dT associated fraction (blue) from U2OS cell fractions. (**F**) The 496 proteins that were identified in both (1) the oligo-dT affinity purification and (2) one of the two crude-polysome fractions, divided up into different functional classes (see Supplementary Table S1 for the complete list). (G-H) Analysis of the ER/cytosolic distribution of various proteins in the crude ribosome fractions (**G**) and oligo-dT affinity purified fractions (**H**) by immunoblot. (**I**) For all proteins expressed in U2OS cells, a comparison of the total number of peptides identified by mass spectrometry in the oligo-dT chromatography experiments (both ER and cytosol; *y-axis*) was plotted against the estimated level of proteins (*x-axis*; data from (15)). Glycolytic enzymes are labeled in red, and ribosomal proteins in magenta. Proteins that did not appear in the oligo-dT chromatography experiments were set to 0.1 peptides, while those proteins that did not appear in the analysis of protein levels were set to 100 molecules/cell. (J-L) For each protein present in either the crude polysome or oligo-dT purifications, the total number of peptides (*y-axis*) identified by mass spectrometry was plotted against the percentage of peptides found in the ER [100% × peptides in ER/(peptides in ER + peptides in cytoplasm); ‘% on ER’ – *x-axis*]. This analysis was performed on the RNA-dependent (**J**) and ribosome-bound (**K**) fractions of the crude polysome preparations, and on the oligo-dT associated proteins (**L**). Note that the *x-axis* is the average of 5 biological replicates for (J, K) and two biological replicates for (L), while the *y-axis* is the total sum of peptides in all experiments (J–L). Classes of proteins that are enriched in either the ER or the cytoplasm are highlighted—carbohydrate metabolic proteins (red), eIFs (yellow) and cytoskeletal-associated proteins (green).

For crude ribosome fractionation, 0.5ml of the C1 or P2 fractions were layered over a 0.5 ml Phy-sucrose buffer (Phy buffer containing 1 M sucrose), and centrifuged at 90 000 RPM for 40 min in a TLA120.2 rotor, to produce a suspension (non-ribosomes) and a pellet (ribosomes). The polysome fraction was then resuspended in 100 μl of Phy Buffer supplemented with 10 μl RNases and incubated for 10 min at room temperature. Treated fractions were centrifuged 60 000 RPM for 1 h to produce a suspension (mRNA-dependent interactors) and pellet (Ribosome- proteins) (see Figure 1B).

For the oligo-dT affinity chromatography 0.5 ml of C1 or P2 fractions were incubated with an equal volume of a 50% slurry of oligo(dT) beads (NEB #S1408S) in Phy buffer, or unconjugated Protein A beads (Thermo Fisher Cat#101041) overnight at 4°C. Beads were washed 5 times with 1 ml cold Phy buffer. Proteins were eluted off the beads by incubating them with 2× Laemmli buffer at 65°C for 5 min.

### Polysome analysis by sucrose centrifugation

18 × 10^6^ U2OS were treated with cycloheximide (100 μg/ml) or Homoharringtonine for 10 min. Cells were collected via trypsinization and sedimented at 800 *g* and washed with ice cold PBS (supplemented with 100 μg/ml cycloheximide) three times. Cells were resuspended in 1 ml polysome lysis buffer (20 mM HEPES–KOH pH 7.4, 5 mM MgCl_2_, 100 mM KCl, 1% Triton X-100, 100 μg/ml cycloheximide) supplemented with 20 U/ml Superase RNAse Inhibitor (Thermo Fisher Cat# AM2694), and protease inhibitor cocktail (Roche). Lysates were cleared via centrifugation at 16 000 *g* for 10 min. 500 μl of lysate saved for RNA input or total. The remaining 500 μl was layered on a 20–60% sucrose gradient buffer (20 mM HEPES–KOH pH 7.4, 5 mM MgCl_2_, 100 mM KCl, 100 μg/ml cycloheximide, and either 20% or 60% sucrose weight/volume) generated using the Biocomp Gradient Master. Lysates centrifuged at 36 000 *g* for 2 h in a SW-41 rotor. Samples were collected and OD260 was continuously measured using the Biocomp Piston Gradient Fractionator. For RNA-seq analysis, polysome fractions (disomes and heavier) were pooled together and RNA was extracted using Trizol-LS (Thermofisher Cat#10296010) protocol. For total fraction, RNA was extracted from saved input. For immunoblotting, proteins from individual fractions were salted out via a TCA precipitation, washed in 100% acetone and re-suspended in 5× Laemmli buffer.

### Ribosome co-sedimentation

18 × 10^6^ U2OS cells were pretreated with either cycloheximide (100 μg/ml) for 10 min or puromycin (200 μM) for 30 min and then collected by trypsinization. The cells were washed twice in ice cold PBS and lysed in ribosome lysis buffer (125 mM KCl, 5 mM MgCl_2_, 20 mM HEPES–KOH pH 7.4, 250 mM sucrose, 0.08% digitonin, 100 μg/ml cycloheximide). Unlysed cells were removed by centrifugation at 800 *g* for 10 min, and the resultant supernatant was centrifuged for 16 000 *g* to remove cellular debris. For ADP, ATP, PEP and F-1,6-BP ribosome sedimentations, the indicated amount of metabolite was added to cleared lysate. The concentration of KCl in the cleared supernatant was adjusted to 500 mM for high salt conditions or remained at 125 mM for physiological conditions. 0.5 ml of cleared supernatant was layered on 0.5 ml of either a high salt (500 mM KCl) or low salt (125 mM KCl) sucrose cushion (1 M sucrose, 5 mM MgCl_2,_ 20 mM HEPES–KOH pH 7.4, 100 μg/ml cycloheximide) in a 1 ml polycarbonate tube and then centrifuged at 90 000 RPM for 1 hour in a TLA-120.2 rotor. The pellet was washed twice in ice cold dH_2_O prior to solubilization in suspension buffer (125 mM KCl, 5 mM MgCl_2_, 20 mM HEPES–KOH pH 7.4).

### Salt-washed ribosome isolation and In vitro co-sedimentation

To generate salt washed ribosomes, 500 ml of HEK293F cells were collected via centrifugation at 800 *g*, and washed 5 times with ice cold PBS. Cells were lysed in modified ribosome lysis buffer (125 mM KCl, 5 mM MgCl_2_, 50 mM Tris–HCl pH 7.4, 250 mM sucrose, 1% NP-40). Lysates were cleared at 16 000 *g* for 10 min. The concentration of KCl in the cleared supernatant was adjusted to 500 mM KCl. 0.5 ml of cleared supernatant was layered on 0.5 ml of high salt (500 mM KCl) sucrose cushion (1 M sucrose, 5 mM MgCl_2,_ 50 mM Tris–HCl pH 7.4) in a 1 ml polycarbonate tube and then centrifuged at 90 000 RPM for 1 h in a TLA-120.2 rotor. The pellets were re-suspended in modified suspension buffer (500 mM KCl, 5 mM MgCl_2,_ 50 mM Tris–HCl pH 7.4). Re-suspended pellets were subjected to an additional round of centrifugation—layering 0.5 ml of the re-suspended pellet solution over 0.5 ml of high salt cushion buffer in a 1 ml polycarbonate tube and then centrifuged at 90 000 RPM for 1 h in a TLA-120.2 rotor. Pellets were then re-suspended in suspension buffer (25 mM KCl, 5 mM MgCl_2,_ 50 mM Tris–HCl pH 7.4). Bicinchoninic acid (BCA) assay was used to measure relative ribosome concentrations. Equal amounts of salt-washed ribosomes, were mixed with equal molar amounts of either recombinant GST or GST-PKM1 in a 0.1 ml suspension buffer and incubated on ice for 30 min. This binding solution was layered on a 0.5 ml sucrose cushion (1 M sucrose, 5 mM MgCl_2,_ 50 mM Tris–HCl pH 7.4) in a 1 ml polycarbonate tube and then centrifuged at 90 000 RPM for 1 hour in a TLA-120.2 rotor. The supernatant was discarded and the pellet washed twice in ice-cold water. The pellet was re-suspended in 1× Laemmli buffer and denatured at 95°C for 5 min.

### Microscale thermophoresis

Purified PKM1 was fluorescently labeled with fluorescein-5-ex succinimidyl ester (Invitrogen) on lysine residues according to the manufacturer's protocol by mixing PKM1 at a final concentration of 3.5 μM protein with an 8-fold molar excess of dye at room temperature for 30 min in the dark. Free dye was eliminated through extensive dialysis and an 80 nM stock solution of labeled PKM in MST buffer (PBS + 5mM MgCl_2_) was prepared.

Prior to mixing, PKM1 and salt-washed ribosome stock solutions were spun at 21 000 *g* for 5 min to remove any aggregated species. We then performed a 1:1 serial dilution of purified ribosomes in MST buffer. All dilutions were performed in 200 μl PCR strips (Starstedt). Purified ribosome dilutions were mixed 1:1 with labeled PKM1, yielding a final PKM1 concentration of 40 nM and a ribosome concentration range from 0 to ∼1.5 μM. Binding reactions were incubated for 30 min before loading into standard glass capillaries (NanoTemper Technologies). MST measurements were performed on a Monolith NT.115 microscale thermophoresis instrument (NanoTemper Technologies) at room temperature using a BLUE LED power of 60% and a medium MST-IR laser power. The resulting dose response curves from three biological replicates were exported from the NanoTemper Technologies Analysis Software (Version 2.3) into GraphPad Prism 6 where the *K*_d_ was calculated by globally fitting the data from the three curves to a sigmodal dose response assuming a 1:1 binding model.

### Radio immunoprecipitation

8.8 × 10^6^ U2OS cells were pulsed with 25 μCi of Cys/Met-protein label (Perkin-Elmer Cat#NEG772002MC) for 15 min. Cells were lysed using 1 ml radio-IP lysis buffer (20 mM HEPES–KOH pH7.4, 150 mM NaCl, 100 mM iodoacetamide, protease inhibitor tablet, 1% NP-40). The lysate was cleared at 16 000 *g* for 10 min. Cleared lysates were incubated with either 4 μg of anti-Tubulin (mouse monoclonal DM1A, 1:5000, Sigma) or anti-G3BP2 (Proteintech Cat#16276-1-AP) antibody for 30 min. 50 μl of protein-A slurry was added to lysates and were shaken overnight at 4°C. After immunoprecipitation, protein-A beads were washed 4 times using radio-IP wash buffer (20 mM HEPES–KOH pH 7.4, 150 mM NaCl, 1% NP-40). IP samples were denatured, on beads, by the addition of 50 μl 5× Laemmli buffer and by heating at 95°C for 5 min. Samples were run through a 10% SDS-PAGE. Gel was fixed via a 15-min incubation in fixing solution (50% MeOH, 10% acetic acid, 40% dH_2_O). The fixed gel was dried on Wattman paper and exposed on a phosphor screen for ∼1 week. Images were taken with a Typhoon-FLA 9000 imager.

### Immunoblot analysis

Samples were denatured with Laemmli sample buffer at 65°C for 5 min and separated by SDS-PAGE on 6–15% polyacrylamide gels. Separated proteins were transferred to nitrocellulose, stained by ponceau for general quality control and immunoprobed with antibodies as following: Aldolase-A (rabbit polyclonal, 1:1000, Cell Signaling Technology), Aly (rabbit polyclonal (37), 1:1000, Sigma), α-Tubulin (mouse monoclonal DM1A, 1:5000, Sigma), β-Actin (mouse monoclonal, 1:1000, Sigma), eIF3A (rabbit polyclonal, 1:1000, Novus), eIF3B (rabbit polyclonal, 1:1000, Bethyl), eIF3C (rabbit polyclonal, 1:1000, Bethyl), eIF3D (rabbit polyclonal, 1;1000, Bethyl), eIF3E (rabbit polyclonal, 1:1000, Bethyl), eIF3F (rabbit polyclonal, 1:1000, Bethyl), eIF3G (rabbit polyclonal, 1:1000, Bethyl), eIF3I (mouse monoclonal, 1:1000, Biolegend), eIF3K (rabbit polyclonal, 1:1000, Bethyl), eIF4A2 (rabbit polyclonal, 1:1000, Abcam), eIF4B (rabbit polyclonal, 1:1000, Signalway Antibody), eIF4E (rabbit polyclonal, 1:1000, Signalway Antibody), eIF4E2 (mouse monoclonal, 1:1000, Santa Cruz Antibody), eIF4G1 (rabbit polyclonal, 1:1000, Cell Signaling Technology), eIFG2 (rabbit polyclonal, 1:1000, Cell Signaling Technology), Enolase-1 (rabbit polyclonal, 1:1000, Cell Signaling Technology), G3BP1 (rabbit polyclonal, 1:1000, Signalway Antibody), G3BP2 (rabbit polyclonal, 1:1000, Proteintech), GAPDH (rabbit polyclonal, 1:1000, Abgent), Kinectin (rabbit polyclonal, 1:1000, Sigma), RRBP1/p180 (rabbit polyclonal, 1:1000, Sigma), PABP (rabbit polyclonal, 1:1000, Abcam), PFKB2 (mouse monoclonal, 1:1000, Cell Signaling Technology), PFKB3 (rabbit monoclonal, 1:1000, Cell Signaling Technology), PFKL (rabbit polyclonal, 1:1000, Cell Signaling Technology), PGAM1 (mouse monoclonal, 1:1000, Cell Signaling Technology), PKM (rabbit polyclonal, 1:1000, Cell Signaling Technology), RPL4 (mouse monoclonal, 1:1000, Novus), RPS6 (rabbit polyclonal, 1:1000, Cell Signaling Technology), Phospho-S6 Kinase (rabbit polyclonal, 1:1000, Cell Signaling Technology), Sec61α (rabbit polyclonal, 1:1000, Sigma), Sec61β (rabbit polyclonal (38), 1:5000), Trap-α (rabbit polyclonal (38), 1:5000), and UPF1 (goat polyclonal, 1:1000, Abcam).

### Mass spectrometry

Protein samples were separated by electrophoresis on a 10% SDS-PAGE gels and stained with Coomassie brilliant blue staining (BioBasic). Each lane on the gel was cut into 16 samples and destained using 50% acetonitrile with 0.2% ammonium bicarbonate. The gel pieces were shrunk with acetonitrile and air dried. The gel pieces were incubated in digestion mixture of trypsin (10 ng/ul) in 50mM ammonium bicarbonate and incubated at 37⁰C overnight. The following day the gel pieces were centrifuged and the digestion mixture removed. The gel pieces were soaked in 1% formic acid in 50% acetonitrile: 49% water for 15 min, centrifuged and the supernatant combined with the digestion mixture. The samples were lyophilized and resuspended in 50mM ammonium bicarbonate and analyzed by liquid chromatograpy-tandem MS using either an LTQ-XL linear ion-trap mass spectrometer (Thermo Fisher Scientific) or Proxeon Easy-nLC 1200 pump in-line with a hybrid LTQ-Orbitrap velos mass spectromter (Thermo Fisher Scientific). Raw files from LTQ-XL mass spectrometer were uploaded to Prohits database (39) and converted to MGF. The data was analyzed and searched using Mascot (2.3.02; Matrix Science). Raw files from LTQ-Orbitrap mass spectrometer was uploaded to Prohits (39) and converted to mzXML. The data was analyzed and searched using X!Tandem (40). Raw files available on MassIVE (MSV000090941) and proteomeXchange (PXD038978).

We analyzed ER and cytosolic fractions from five crude polysomes preparations (both RNA-dependent and ribosome-bound fractions) and two oligo-dT affinity purifications by mass spectrometry analysis. Note that due to the lower overall levels of protein in the Mock Bead pulldowns (Figure 1D), the peptide counts in this fraction are likely to be over-sampled in comparison to the oligo-dT purified samples. We scored proteins as present in the ribosome and RNA-dependent fractions if represented by peptides in at least two of three separate biological replicates. For the oligo-dT experiments, proteins had to contain peptides that appeared in two of three biological replicates and be at least two-fold enriched over the mock bead pulldown in both experiments. Manual curation removed 30 proteins that were likely contaminants (e.g. Keratin, RNase, Albumin, mitochondrial proteins). Percent ER was calculated by determining the fractional representation of peptides from a protein in a given pool (pools included: ER RNA-dependent, Cyto RNA-dependent, ER Ribosome-bound, Cyto Ribosome-bound, ER mRNP(oligo-dT)-bound, Cyto mRNP(oligo-dT)-bound) and averaging these between experiments.

### eCLIP sequencing library preparation

Enhanced crosslinking and immunoprecipitation (eCLIP) was performed on HepG2 cells in biological duplicates, essentially as described (41). For each replicate, extract from 20 × 106 UV-crosslinked (254 nm, 400 mJ/cm2) HepG2 cells was prepared by sonication in 1 ml lysis buffer, treated with RNase I (40 U, LifeTech), and immunoprecipitated overnight at 4°C with 2 μg affinity-purified rabbit anti-PKM2 antibody raised against a C-terminal peptide (Sigma cat. # SAB4200105, Lot #030M4874) pre-coupled to Dynabeads sheep M-280 anti-rabbit IgG beads (LifeTech). Prior to IP, a 20 μl aliquot of extract was removed and stored at 4°C for preparation of the size-matched input (SMInput) control. To ensure IP stringency, a series of wash steps was employed as follows: 2x low stringency wash buffer (20 mM Tris–HCl, pH 7.4, 10 mM MgCl2, 0.2% Tween-20), 2x high stringency buffer (15 mM Tris–HCl pH 7.4, 5mM EDTA, 2.5 mM EGTA, 1% Triton-X 100, 1% sodium deoxycholate, 0.1% SDS, 120 mM NaCl, 25 mM KCl), 2x high salt wash buffer (50 mM Tris–HCl, pH 7.4, 1M NaCl, 1mM EDTA, 1% NP-40, 0.1% SDS, 0.5% sodium deoxycholate), 2x low stringency wash buffer, and 2x PNK buffer (50 mM Tris–HCl pH 7.4, 10mM MgCl2, 0.5% NP-40). After washing, immunoprecipitated protein-RNA complexes were dephosphorylated and 3′-linker ligated on-bead to a custom oligonucleotide adapter. All samples (IPs and SMInputs) were heated in LDS Sample Buffer (LifeTech, 70°C, 10 min) and run on 4–12% NuPAGE polyacrylamide gels in MOPS running buffer (LifeTech). Complexes were wet-transferred to iBLOT nitrocellulose membranes in NuPAGE transfer buffer (all LifeTech) containing 10% methanol (overnight, 4°C, 30V). Immunoprecipitation was confirmed by performing standard immunoblotting on a fraction of all samples. RNA-protein complexes in the range from 75 kDa (the apparent molecular mass of PKM2) to 135 kDa (corresponding to PKM2-crosslinked RNAs of ∼220 nucleotides in length) were excised from the membrane. RNA was released by proteinase K treatment in urea and recovered by phenol chloroform extraction and column purification (RNA Clean-Up kit; Zymo Research). Input samples were dephosphorylated and 3′-linker ligated to a custom oligonucleotide primer and all samples (IPs and SMInputs) reverse transcribed using AffinityScript reverse transcriptase (Agilent). After treatment with ExoSAP-IT (Affymetrix) and alkali, cDNAs were recovered by purification on Dynabeads MyONE Silane beads (LifeTech), 5′-linker ligated on-bead to a custom oligonucleotide primer, purified, and recovered in 27 μl. cDNA was quantified by qPCR analysis of a fraction each sample using oligonucleotide primers specific to the 5′ and 3′ adapters. Half of the recovered cDNA was PCR-amplified (Q5 polymerase, NEB) using custom sequence-indexed oligonucleotide primers with the following cycle numbers: replicate 1: input 8, IP 15; replicate 2: input 8, IP 13. PCR products were purified (Agencourt AMPure XP beads; Beckman Coulter), size-selected to 175 -350 bp on 2% agarose gels, extracted (MinElute Gel Extraction kit; Qiagen), and quantified on a TapeStation using D1000 ScreenTape (Agilent). Libraries were sequenced on a HiSeq 4000 instrument (Illumina) in paired-end 55 bp mode. All sequence data was deposited to GEO (GSE229167).

### Computational analysis of eCLIP-seq data

#### Trimming and mapping

Sequencing reads were processed essentially as described (42). Reads were adapter trimmed and mapped to human-specific repetitive elements from RepBase (version 18.04) by STAR (43). Repeat-mapping reads were removed and remaining reads mapped to the human genome assembly hg19 with STAR. PCR duplicate reads were removed using the unique molecular identifier (UMI) sequences in the 5′ adapter and remaining reads retained as ‘usable reads’. Peaks were called on the usable reads by CLIPper (44) and assigned to gene regions annotated in Gencode (v19). Each peak was normalized to the sizematched input (SMInput) by calculating the fraction of the number of usable reads from immunoprecipitation to that of the usable reads from the SMInput. Peaks were deemed significant at >8-fold enrichment and *P*-value < 10^−5^ (Chi-square test). All sequencing and processing statistics are in Supplementary Table S3.

#### Irreproducibility discovery rate

To test the quality of the datasets, Irreproducible Discovery Rate (IDR) analysis as described in (45) was performed, yielding 1907 common peaks between the two replicates (consistency ratio: 5.37; rescue ratio: 2.40).

### Radiolabeling of PKM2-bound RNA fragments

Immunoprecipitation and end-labeling was performed as described (46) with modifications. 20 million UV crosslinked HepG2 cells were lysed in 550 μl lysis buffer (50 mM Tris–HCl pH 7.4, 100 mM NaCl, 1% NP-40, 0.1% SDS, 0.5% sodium deoxycholate) with protease inhibitor cocktail (Roche). Lysates were sonicated for 5 min (BioRuptor, low setting, 30 s on/off) in an ice-cold water bath. After addition of 2.2 μl Turbo DNase (NEB) and 5.5 μl RNase A (Millipore cat. #20-297), diluted 1:100 (high RNase) or 1:10000 (low RNase) in low-stringency wash buffer (20 mM Tris–HCl, pH 7.4, 10 mM MgCl_2_, 0.2% Tween-20), samples were incubated at 37°C for 5 min with shaking. RNase digestion was stopped with 11 μl Murine RNase inhibitor (NEB) and insoluble material removed by centrifugation (15 min, 15 000 g, 4°C). Protein-RNA complexes were immunoprecipitated overnight at 4°C with PKM2 antibody (SAB4200105, Sigma) or normal rabbit IgG (Thermo Fisher) pre-coupled to magnetic beads (Dynabeads M-280 Sheep anti-Rabbit IgG, Thermo Fisher). A series of wash steps was employed to ensure stringency, as follows: 2× low stringency wash buffer (see above), 2x high stringency buffer (15 mM Tris–HCl pH 7.4, 5mM EDTA, 2.5 mM EGTA, 1% Triton-X 100, 1% sodium deoxycholate, 0.1% SDS, 120 mM NaCl, 25 mM KCl), 2× high salt wash buffer (50 mM Tris–HCl, pH 7.4, 1 M NaCl, 1mM EDTA, 1% NP-40, 0.1% SDS, 0.5% sodium deoxycholate), 2× low stringency wash buffer, and 2× PNK buffer (50 mM Tris–HCl pH 7.4, 10mM MgCl_2_, 0.5% NP-40). Protein–RNA complexes were radiolabeled on-bead in 40 μl reactions with T4 polynucleotide kinase (NEB) and 2 μl [γ-32P]ATP (6000 Ci/mmol, 10 mCi/ml) for 10 min at 37°C. Beads were washed 3x in PNK buffer, resuspended in NuPAGE LDS Sample Buffer (Thermo Fisher) containing 0.1 M DTT. Protein-RNA complexes were denatured at 75°C for 15 mins and run on 4–12% NuPAGE Bis-Tris gels in NuPAGE MOPS running buffer (all Thermo Fisher) at 150 V for 1.5 h, wet-transferred to nitrocellulose membrane using NuPAGE transfer buffer (Thermo Fisher) with 10% methanol for 3h at 200 mA. The membrane was exposed to film for 20 min at room temperature and the film developed.

### Ribosome footprint profiling

HEK293T cell line was obtained from ATCC and maintained in Dulbecco's modified Eagle's medium supplemented with 10% fetal bovine serum. Cells were washed twice with 10 ml of ice-cold PBS and placed on ice. Lysis was carried out on plates using 400 μl volume (Tris–HCl 20 mM pH 7.4, 150 mM NaCl, 5 mM MgCl_2_, 1 mM DTT, 100 μg/ml cycloheximide, 1% Triton-X). 5 μl of RNaseI (Invitrogen AM2249) was added to the lysates, followed by a 1 h digestion at 4°C. Immediately following digestion, ribonucleoside vanadyl complex (NEB S1402S) (20 mM) was added to inhibit further digestion. Digested lysates (400 μl each) were loaded on a sucrose cushion (20 mM Tris–Cl titrated to pH 7.4, 150 mM NaCl, 5 mM MgCl_2_, 1% TritonX-100, 34% sucrose, 1 mM DTT, 100 ug/ml cycloheximide) and centrifuged in a SW41Ti rotor (Beckman Coulter) at 38000 RPM at 4°C for 2.5 h to pellet ribosomes. 700 μl QIAzol Lysis Reagent (Qiagen) was used to resuspend the ribosome pellet, which was was transferred to a fresh 1.5 ml tube and frozen until further processing. To extract RNA, samples were thawed at room temperature and processed with the Qiagen miRNeasy Kit per manufacturer's instructions. RNA was eluted in 20 μl nuclease-free water and size-selected by electrophoresis of 3 μg inputs in a Novex denaturing 15% polyacrylamide TBE–urea gel. The 26–34 nt RNA fragments were excised, crushed with a pestle, and extracted in 310 μl gel extraction buffer (300 mM NaOAc pH 5.2, 1 mM EDTA, 0.25% w/v SDS) by incubating on dry ice for 30 min then overnight at room temperature. The gel-liquid mixture was passed through a Spin-X filter (Corning 8160) and the elution was precipitated with 1.5 μl Glycoblue (5 mM MgCl_2_, 75% ethanol). RNA was pelleted by centrifugation at 23 300 *g* at 4°C for 1 h and resuspended in 10 μl nuclease-free water. 25 ng inputs of size-selected ribosome footprints were immediately processed for library preparation using the D-Plex Small RNA-Seq Kit (Diagenode) per manufacturer's instructions, with some modifications. Approximately half of the resulting cDNA (14 μl) was amplified for 9 cycles. Following quantitation of the target libraries with Agilent HS DNA Kit and Bioanalyzer, the resulting libraries were pooled to equimolar concentrations and cleaned with AMPure XP beads at 1.8x concentration (Beckman Coulter) and eluted in 30 μl TE. To enrich for ribosome footprints in the libraries, the entire elution was size selected in a 3% agarose precast gel (Sage Science) in the BluePippin system using a 172–206 nt range with tight settings. The resulting libraries were sequenced with NovaSeq SP SE 100 (Illumina). All sequence data was deposited to GEO (GSE202881).

### Quantitative reverse transcription PCR (RT-qPCR)

PKM depletion was verified using RT-qPCR. Six days post-lentiviral infection, PKM shRNA and control shRNA (2 × 10^5^) treated HEK293T cells were harvested by TRIzol (Zymo Research). RNA isolated was carried out using Direct-zol RNA miniprep kit (Zymo Research). 450 ng of total RNA was used for synthesis of cDNA by Superscript Reverse transcriptase IV (Invitrogen) using random hexamers (Invitrogen). Resulting cDNA was diluted 1:5 prior to use. qPCR was carried out using Power SYBR Green master mix and 200 nM of oligos (using GAPDH as an internal control, Forward Primer: GAATGACCCCTTCATTGACC, Reverse Primer: TTGATTTTGGAGGGATCTCG). Two unique pairs of primers were used for analyzing the depletion of each PKM isoform (PKM1 Forward Primer: TCACTCCACAGACCTCATGG, PKM1 Reverse Primer: GAAGATGCCACGGTACAGGT, PKM2 Forward Primer: ATCGTCCTCACCAAGTCTGG, PKM2 Reverse Primer: GAAGATGCCACGGTACAGGT). The ViiA 7 Real-Time PCR system was used as follows: 50°C for 2 min, 95°C for 2 min and 40 cycles at 95°C for 1 min and 60°C for 30 s followed by melt curve analysis. The data was analyzed using the 2^–ΔΔCt^ method. The experiment was carried out in three technical replicates.

### RNA sequencing

To extract RNA, samples were thawed at room temperature and processed with the Qiagen miRNeasy Kit per manufacturer's instructions. RNA was eluted in 25 μl nuclease-free water and processed with the CATS RNA-seq Kit v2 per manufacturer's instructions (Diagenode C05010045). Approximately half of the resulting cDNA (14 μl) was amplified for 11 cycles. PCR products were cleaned as outlined in the Diagenode protocol and the resulting libraries were sequenced with NovaSeq SP SE 100 (Illumina). All sequence data was deposited to GEO (GSE202881).

### Computational analyses of ribosome profiling data

Ribosome profiling data were processed using RiboFlow (47). We extracted the first 12 nucleotides from the 5′ end of the reads using UMI-tools (48) with the following parameters: ‘umi_tools extract -p ‘^(?P < umi_1 > .{12})(?P < discard_1 > .{4}).+$’ –extract-method = regex’. The four nucleotides downstream of the UMIs are discarded as they are incorporated during the reverse transcription step. Next, we used cutadapt (49) to clip the 3′ adapter AAAAAAAAAACAAAAAAAAAA. After UMI extraction and adapter trimming, ribosomal and transfer RNAs were filtered by alignment using Bowtie2. The remaining reads were mapped to human transcriptome and alignments with mapping quality greater than two were retained. UMIs were used for deduplication and .ribo files are created using RiboPy (47). Identification of differential ribosome occupancy and RNA expression was carried out as previously described (50,51).

### Pause site detection

P-site adjustment was carried out using the metagene plots to determine offsets as a function of read length. We selected genes for which normalized CDS coverage is equal to or larger than one in all experimental replicates. We then fitted negative binomial distributions for each genes’ ribosome occupancy coverage vector at nucleotide resolution after removing 5% top and bottom outliers. Using these estimated parameters, we calculated *P*-values for each nucleotide resolution that captures the probability of the observed read counts are derived from this distribution. We the combined *P*-values from experimental replicates with Fisher's method. Finally, positions with a combined *P*-value < 1 × 10^−7^ were defined as putative pause sites.

### Detection of differential pause sites

To find differential pause sites, we compared coverage of the pause sites between shPKM and shCtrl set with edgeR (52) and filtered pause sites with an adjusted *P*-value threshold of 0.05. Some of these pause sites can potentially be attributed to the overall ribosome occupancy coverage difference between shPKM and shCtrl treatments. To filter out such cases, we determined pause sites that were detected in genes with differential ribosome occupancy. We then removed pause sites where the absolute value of the difference between a pause site's log2 fold-change and the log2 fold-change of the gene's ribosome occupancy was <0.8.

### Ribosome foot printing northern blots

16 million U2OS cells were pretreated with cycloheximide (100 μg/ml) for 10 min and then collected by trypsinization. Cells were washed twice in ice cold PBS followed by the addition of 800 μl lysis buffer (20 mM Tris–HCl pH 7.4, 150 mM NaCl, 5 mM MgCl_2_, 1 mM DTT, 100 μg/ml cycloheximide, 1% TritonX). Cells were lysed on ice by vigorous pipetting for 10 min. Lysed cells were centrifuged for 10 min at 800 *g* and the collected supernatant centrifuged for 10 min at 16 000 *g*. The resulting supernatants were treated with 14 μl of RNaseI (Invitrogen AM2294) for 1 h at 4°C to degrade unprotected mRNA fragments. RNA digestion was inhibited by addition of 20 mM ribonucleoside vanadyl complex (NEB S1402S). 500 μl of RNAse digested samples was layered on 500 μl sucrose cushion (1 M sucrose, 5 mM MgCl_2_, 20 mM Tris–HCl pH 7.4, 100 μg/ml cycloheximide) in a 1 ml polycarbonate tube and then centrifuged at 90 000 RPM for 1 h in a TLA-120.2 rotor. Resulting pellet was washed twice in ice cold dH_2_O prior to extraction of RNA from ribosome pellet by TRIzol (ThermoFisher 15596026).

Radiolabelled probes were generated by phosphorylating primers (antisense to our hybridization sites) with [γ^32^P]ATP using T4 polynucleotide kinase (NEB M0201S). Probes were then cleaned using Cytiva G-25 MicroSpin Columns (Cytiva 27532501). Extracted RNA was separated on a 15% UREA-PAGE gel, 10 μg per well, in 0.5X TBE buffer. RNA was transferred by electroblotting onto charged nylon membrane (Cytiva Amersham Hybond-N+) as per electroblot manufacturers specification (Idea Scientific, catalogue 4003). Transferred RNA was crosslinked to membrane by UV (Autocrosslink, UV Stratalinker Model 1800), and baked at 80°C for 30 min. Membranes were incubated and rotated in 50 ml pre-hybridization buffer (5× Saline Sodium Citrate (SSC) buffer, 20 mM Na_2_HPO_4_ pH 7.2, 7% SDS, 2X Denhardt's Solution, Sheared Salmon Sperm DNA [1 mg per blot]) for at least 2 h at 30°C in hybridization oven prior to the addition of radiolabelled probes. Membranes incubated with hot hybridization solution overnight at 30°C in hybridization oven. After hybridization, membranes washed thrice, 50 ml wash buffer (3× SSC, 25 mM Na_2_HPO_4_, pH 7.2, 5% SDS), 30 min each at 30°C. Washed membranes were exposed on phosphor screen. Images were taken with a Typhoon-FLA 9000 imager.

### Nascent polypeptide analysis

Nascent polypeptides were isolated by adapting the protocol from Aviner et al. (53). 50 million U2OS cells were collected by trypsinization. Cells washed twice with ice-cold PBS and re-suspended in 1 ml PUNCH-P polysome buffer (50 mM HEPES pH 7.5, 10 mM MgCl_2_, 25 mM KCl, supplemented with protease inhibitor cocktail [Roche] and RNase inhibitor [AM2694]). 120 ul of lysis buffer (11% sodium deoxycholate, 11% Triton-X) added to resuspended cells, and incubated on ice for 20 min with occasional pipetting. Cell lysate clarified at 16 000 *g* for 15 min. 500 ul Clarified lysate layered on 500 ul sucrose cushion (PUNCH-P polysome buffer containing 1 M sucrose). Translating ribosomes pelleted by centrifugation at 37 000 RPM for 160 min. Ribosome pellet washed with 500 ul dH_2_O and resuspended in 90 ul polysome buffer. Resuspended ribosomes were supplemented with either 100 pmol of puromycin or biotin-puromycin analogue (Jena Bioscience, Biotin-dC-puromycin) per 1 OD254 of detected ribosomes and incubated at 37°C for 15 min to release nascent chains. 5 ul of each reactions were kept to assess biotinylation efficiency. Streptavidin beads (ThermoFisher, 20347) were added to remaining reaction volumes (∼40 uL) at 5 ul of slurry per 1 OD254 of detected ribosomes to capture nascent chains. Streptavidin-biotin capture was performed overnight at 4°C. After capture, streptavidin beads were washed with 6 times in PUNCH-P polysome buffer. To elute biotinylated proteins, streptavidin beads were incubated with a solution of 95% formamide, 10 mM EDTA, and 25 mM free biotin at 95°C for 10 min. Eluted nascent chains mixed with 5X Laemmli buffer and analyzed by SDS-PAGE.

## RESULTS

### Proteomic analysis of cytosolic and ER polysome-associated factors

The cytosol and ER represent the two major subcompartments where cellular protein synthesis occurs, each containing distinct pools of mRNAs and unique translational regulatory systems (20–28). To determine the spatial distribution of post-transcriptional regulatory factors we isolated polysomes from ER and cytosolic fractions (Figure 1A) from human osteosarcoma (U2OS) cells (Figure 1B). These isolated polysomes were treated with RNase to liberate putative RNA-binding proteins (‘RNA-dependent fraction’), and resedimented to pellet ribosomes and associated proteins (‘Ribosome-bound fraction’). We analyzed the composition of the RNA-dependent and ribosome-bound fractions (Figure 1C) by mass spectrometry, as previously described (26). In parallel we also isolated messenger ribonuclear protein (mRNP) complexes from the ER and cytosol using oligo-dT affinity chromatography (‘mRNP-bound’; Figure 1B, D), or a mock bead control, and analyzed these fractions by mass spectrometry. Our purification conditions, done in the absence of crosslinking, enabled recovery of proteins that are directly and indirectly bound to mRNAs or ribosomes, although we cannot fully rule out the co-purification of other large molecular complexes in our "Ribosome-bound’ fraction.

To ensure that we only classify true interactions, we considered proteins present in (a) the mRNP-bound and (b) either the RNA-dependent or ribosome-bound fractions, as high confidence interactors that associate with either mRNA, or ribosomes. After statistical processing and manual curation (see methods), we identified 496 polysome interactors (Figure 1E, Supplementary Table S1). Nearly all of these proteins (98%; 484/496) had been identified in previous global analyses of proteins that associate with RNA or ribosomes (1–16) (Supplementary Table S2), indicating that our list of polysome interactors is in agreement with the literature.

Our high confidence polysome interactors fell into several broad categories (Figure 1F). These include a wide array of metabolic enzymes, especially those involved in carbohydrate metabolism, similar to what had been uncovered by several genome-wide RNA and ribosome binding protein surveys from a variety of species (1–16,54). The most abundant interactors by spectral counts were glycolytic enzymes, including both spliced isoforms of pyruvate kinase M (PKM1 and PKM2). The detected glycolytic enzymes were not nascent polypeptides emerging from the ribosome, a concern for abundantly expressed proteins, as we found no bias for N-terminal peptides (Supplementary Figure S1), which would be the case for partially synthesized proteins. The presence of full-length glycolytic enzymes (as opposed to partially synthesized polypeptides) in these fractions was further confirmed by immunoblot (Figure 1G, H). Additional glycolytic enzymes that were identified in the mass spectrometry but did not meet our cut-off (e.g. PGAM1, PFKL, PFKB) were also found in the RNA-dependent and ribosome-bound fractions (Figure 1G, H). Lastly, when the number of peptide spectral counts in the oligo-dT pulldown were compared to estimates of overall protein levels in U2OS cells (55), glycolytic enzymes were on par with ribosomal proteins (Figure 1I, glycolytic enzymes are in red, ribosomal proteins are in magenta), suggesting that they were not trace contaminants.

We calculated both the ER and cytosolic distribution of post-transcriptional regulators from our RNA-, ribosome-, and mRNP-bound fractions (Supplementary Table S1) and found that they differed significantly from what would be expected if peptides were randomly assorted (Figure 1J–L, Supplementary Figure S2). As expected, ER-resident proteins were biased towards the ER, whereas ribosomal proteins were not biased suggesting equal sampling of ribosomes from both compartments (Figure 1G, H, Supplementary Figure S3). The ER/cytosolic distribution of individual proteins was highly correlated between the different fractionation protocols (Supplementary Figure S4). Interestingly, eukaryotic translation initiation factors (eIFs) were slightly enriched in the ER for all fractions (Figure 1J–L, yellow data points), and this was verified by immunoblot (Figure 1G). We speculate that this may be due to the presence of non-translating ribosomes that are predominantly in the cytosol. In contrast, cytoskeletal components including actin, tubulin, motor and cytoskeletal-binding proteins were enriched in the cytosol for all fractions (Figure 1J–L, green data points; also see α-tubulin in Figure 1G, H and β-actin in Figure 1H), which may indicate that ribosomes and mRNPs that are transported along cytoskeletal filaments must be disengaged from the ER. Lastly, carbohydrate metabolic enzymes, including glycolytic enzymes, were also enriched in the cytosol (Figure 1J–L, red data points). Indeed, most of these co-sedimented with cytosolic ribosomes in an RNA-dependent manner, suggesting that they bound directly or indirectly to RNA, and this was verified by immunoblot (Figure 1G, H).

### PKM associates with polysomes in a glucose/pyruvate-dependent manner

**Figure 2.**
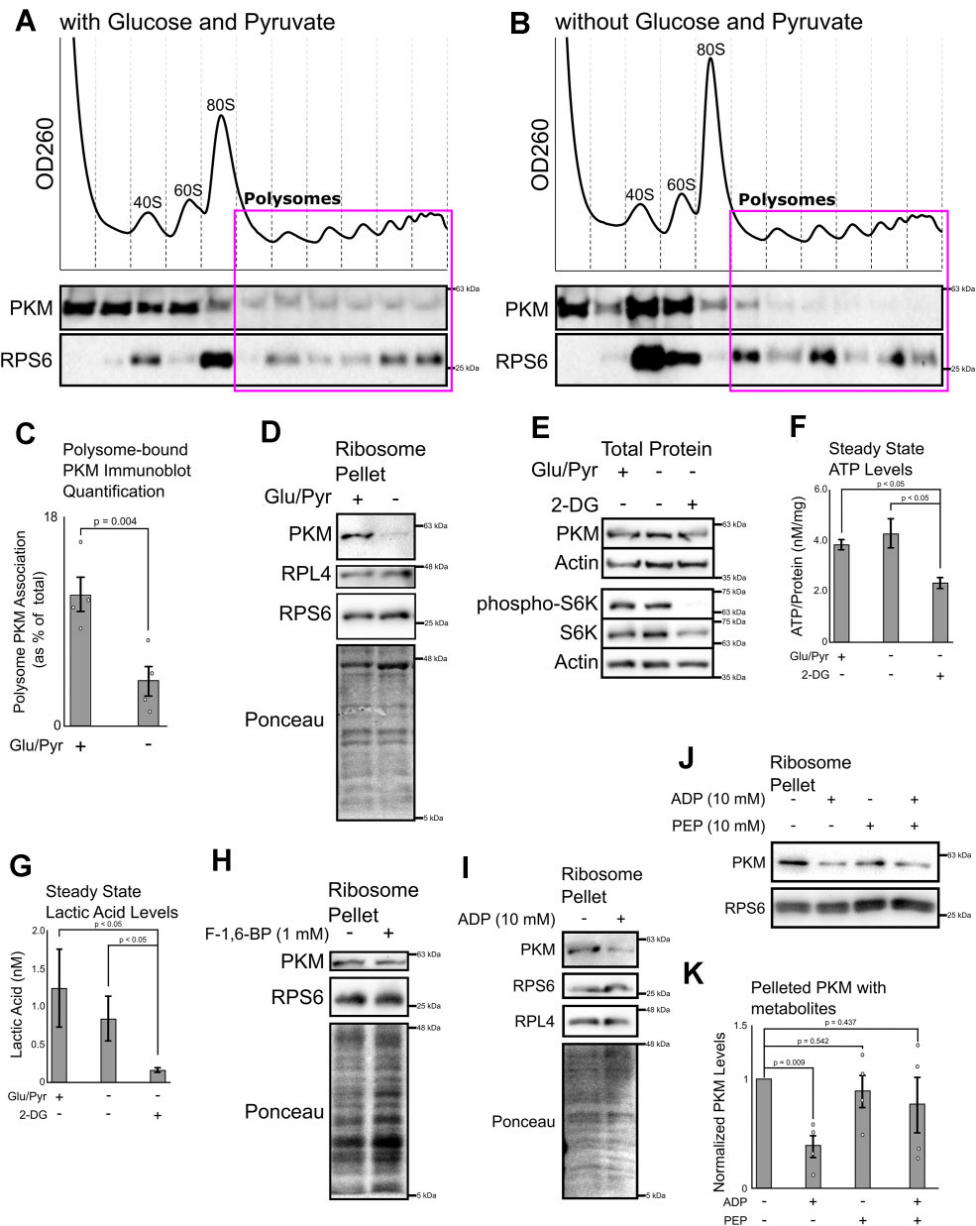
PKM associates with polysomes in a glucose/pyruvate-dependent manner. (A, B) U2OS cells were incubated in DMEM that contained (**A**) or lacked (**B**) glucose (4.5 g/l) and sodium pyruvate (0.11 g/l) for 3 h. 10 min prior to lysis, the cells were treated with 100 μg/ml Cycloheximide to stabilize polysomes. Cell lysates were sedimented through 20–60% linear sucrose gradient at 160 000 *g* for 2 h. Nucleic acids were monitored by OD260, and fractions were monitored by immunoblot for pan-PKM (both PKM1 and 2) and RPS6. (**C**) Quantification of percentage of PKM that associates with the polysome fractions. Each bar represents the mean of four biological replicates plotted alongside standard error, as whiskers, and individual replicates, as dots. *P*-value calculated from paired Student's *t*-test. (**D**) U2OS cells were incubated in DMEM that contained or lacked glucose/pyruvate identical to 1A & 1B. Polysomes were sedimented through a sucrose cushion and probed for pan-PKM and ribosomes (RPL4 and RPS6). Total proteins were also monitored by Ponceau stain. (**E**) U2OS cells were incubated in DMEM that contained or lacked glucose/pyruvate, with or without 2-deoxyglucose (‘2-DG’; 20 mM) for 3 h. Lysates were monitored by immunoblot for pan-PKM, phosphorylated S6 Kinase (p-S6K, a downstream product of mTOR kinase) and Actin. (F, G) U2OS cells were incubated in DMEM that contained or lacked glucose/pyruvate, with or without 2DG for 3 h. Cell lysates were assessed for ATP (**F**) and lactate (**G**) levels. Each bar represents the mean of three biological replicates plotted alongside standard error, as whiskers. *P*-value calculated from paired Student's *t*-test. (H–J) U2OS lysate were spiked with 1 mM fructose-1,6-bisphosphate (**H**) or a combination of either 10 mM PEP, ADP or both (**I**, **J**). Polysomes were sedimented and probed for pan-PKM and ribosomes (RPL4 and/or RPS6). Total proteins were also monitored by Ponceau stain. (**K**) Densitometry analysis of (J). Each bar represents the mean of at least three biological replicates plotted alongside standard error, as whiskers, and individual replicates, as dots. *P*-value calculated from paired Student's *t*-test.

Since we observed that glycolytic enzymes were present in cytosolic polysomes, we asked if their association was sensitive to changes in glycolysis, as the canonical functions of glycolytic enzymes are often regulated by metabolites. We found that PKM, which catalyzes the last step of glycolysis, preferentially co-fractionates with polysomes isolated from cells fed with glucose and pyruvate in comparison to cells starved of these two metabolites for 3 h (Figure 2A–C). PKM-polysome co-fractionation was diminished in U2OS cells pre-treated with homoharringtonine (HHT), which eliminates polysomes, when compared to lysates from cycloheximide-treated cells (Supplementary Figure S5A, B) indicating that PKM migration in the gradient is polysome-dependent. PKM also co-sedimented with isolated ribosomes by sucrose cushion centrifugation in a glucose/pyruvate-dependent manner (Figure 2D). Glucose/pyruvate starvation did not significantly change total levels of PKM, ATP or lactic acid (Figure 2E–G), suggesting that this treatment did not activate major stress pathways in our cells. In contrast, treatment with 2-deoxyglucose (2-DG), an inhibitor of glycolysis, impaired mTOR signaling, as monitored by the phosporylation of S6K, and caused a decrease in the levels of ATP and lactic acid (Figure 2E–G).

The *PKM* gene produces two proteins due to alternative splicing. Since the M2 isoform (PKM2) is expressed at a higher level than the M1 (PKM1) (56), we tested whether PKM sedimentation is affected by the exogenous addition of the M2 specific allosteric regulator fructose-1,6-bisphosphate but found that it had no effect on PKM sedimentation (Figure 2H). As we detected both PKM1 and PKM2 peptides in our cytosolic polysome screen, and given the insensitivity of PKM ribosome interaction to fructose-1,6-bisphosphate, we believe that the PKM ribosome interaction is isoform-independent. Glucose starvation is known to rapidly increase the levels of ADP (57), which directly binds to and is a substrate of PKM. We found that the exogenous addition of ADP to lysate from unstarved cells disrupted the co-sedimentation of PKM with ribosomes (Figure 2I). In contrast, exogenous addition of ATP, also a PKM substrate, significantly increased PKM co-sedimentation with ribosomes although this was highly variable between experiments (Supplementary Figure S5C). The other substrates, pyruvate and phosphoenolpyruvate (PEP), had no effect on PKM pelleting but when PEP was added with ADP there appeared to be a loss in ADP dependent regulation, although this was highly variable between experiments (Figure 2J, K). Overall, these suggests that changes in glycolytic flux, as seen in transient glucose and pyruvate restriction, can modulate PKM-ribosome interactions through changes in ADP, and possibly ATP, levels.

### PKM associates with ribosomes that translate mRNAs coding for glutamate- or lysine-rich tracts

**Figure 3.**
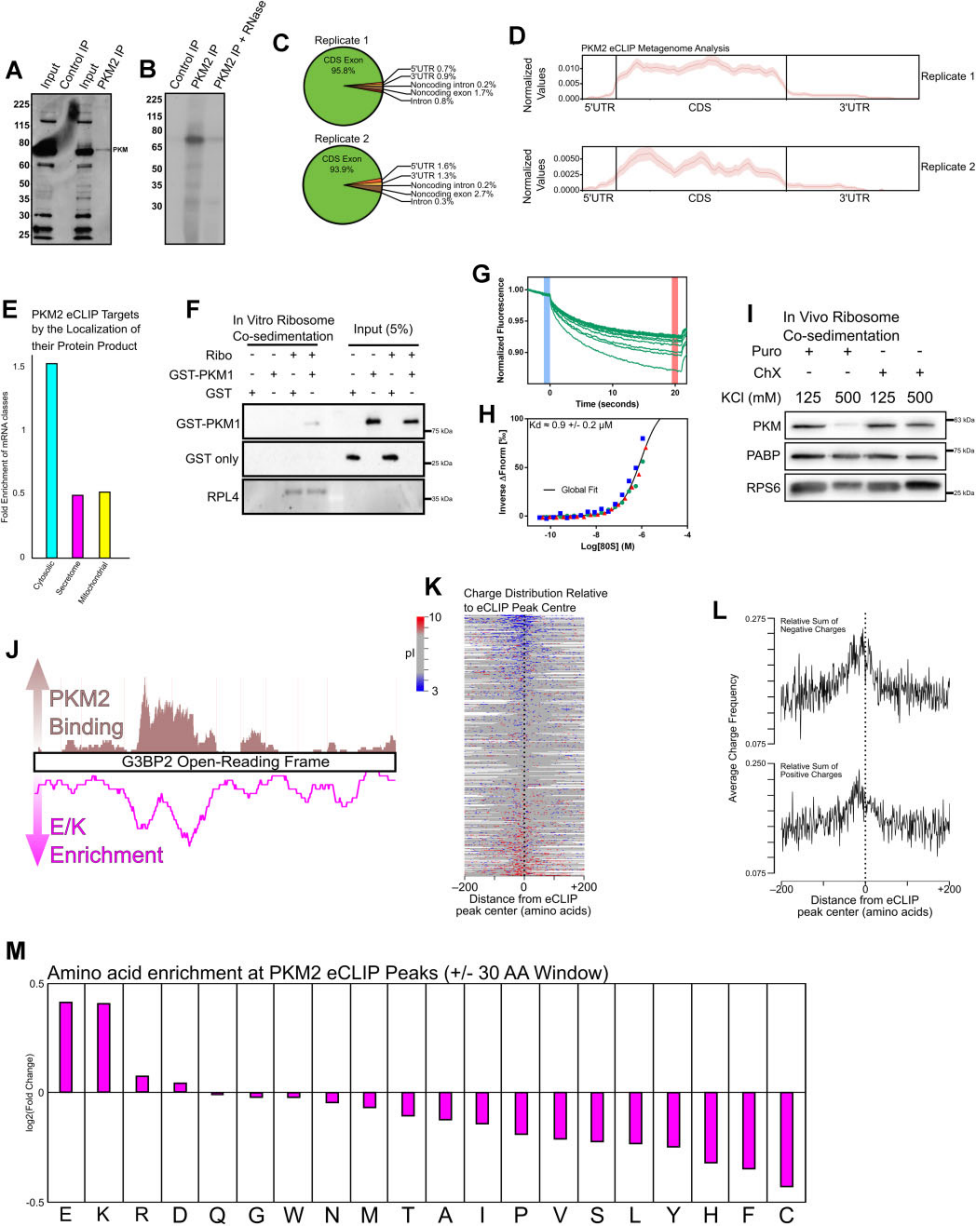
PKM2 associates with the coding sequence of mRNAs encoding cytosolic proteins. (**A**) PKM2 was immunoprecipitated from HepG2 cell lysates, separated by SDS PAGE and immunoblotted for PKM. (**B**) Mock (‘Control IP’) and PKM2-immunoprecipitates (‘PKM2 IP’) were labeled with [γ^32^P]-ATP using polynucleotide kinase. To verify whether the band in question contained labeled RNA, the phosphorylated immunoprecipitate was treated with RNase (‘+ RNase’). The resulting reactions were resolved by SDSPAGE and processed for autoradiography. One major band of ∼72 kDa, corresponding to PKM2, was observed. (**C**) Mapping of PKM2 eCLIP reads (8-fold enrichment, *P*-value < 10^−5^) to various annotated parts of the human transcriptome. (**D**) Metagene plot of PKM2 eCLIP reads along the normalized length of protein coding mRNAs. (**E**) PKM2 eCLIP target mRNAs were analyzed for enrichment of various classes of encoding proteins localized to cytosol & nucleoplasm (cytosol), ER-membrane bound and secreted (secretome), and mitochondria (mitochondrial) compared to background rates found in the human proteome. (**F**) Purified GST or PKM1-GST were incubated with and without salt-washed ribosomes, isolated from HEK293F cells. Incubated reactions were sedimented through a sucrose cushion and were immunoblotted for GST or RPL4. Only 5% of total reaction was loaded as input. (**G**) Representative fluorescence time trace displaying the thermophoretic movement of fluorescein labeled GST-PKM1 bound to increasing concentrations of salt-washed ribosomes. (**H**) Dose-response curve derived from the time traces of three independently purified batches of salt-washed ribosomes globally fit to yield an estimated *K*_d_ of ≈ 0.9 ± 0.2 μM. (**I**) U2OS cells were treated with either 200 μM Puromycin (200 μM) or Cycloheximide (10 μg/ml) for 30 min and then lysed and incubated in isotonic (125 mM KCl) or a high salt (500 mM KCl) buffer. Ribosomes/polysomes were then isolated by centrifugation through a sucrose cushion and the pellets were analyzed by immunoblot for pan-PKM, PABP and RPS6. (**J**) Distribution of eCLIP reads for PKM2 across the *G3BP2* open-reading frame (adapted from UCSC genome browser track: https://s3-us-west-1.amazonaws.com/sauronyeo/20170112_PKM2_CLIP/hub.txt), overlaid with the enrichment of glutamic acid and lysine across the G3BP2 polypeptides (in a 30 amino acid moving average). (**K**) PKM2 eCLIP peaks were aligned (denoted at position ‘0’ with dashed line) and the encoded polypeptides were analyzed for the presence of positive (blue) or negative (red) amino acids. Neutral amino acids are in grey. (**L**) The frequency of negative and positive amino acids with respect to the center of PKM2 eCLIP peaks (denoted at position ‘0’, with dashed line). (**M**) The enrichment of all 20 amino acids within the vicinity of the PKM2 eCLIP peak (±30 amino acid window).

Given that we and others (1,6,10–15,54) have identified PKM as a putative RNA-binding protein, we immunoprecipitated the major isoform, PKM2 (Figure 3A), under very stringent conditions from lysates of UV-crosslinked HepG2 cells, followed by limiting RNase treatment. When these immunoprecipitates were labeled with [γ^32^P]-ATP using polynucleotide kinase, one major RNase-sensitive band, corresponding to PKM2 was observed (Figure 3B), indicating that this protein was in close proximity to endogenous RNAs. Next, we isolated the cross-linked RNAs and analyzed these by eCLIP-Seq (41). We identified approximately 4000 enriched crosslinking peaks in comparison to the size-matched inputs (8-fold enrichment, *P*-value < 10^−5^), distributed over transcripts from 961 genes from two independent replicates (Supplementary Table S3). Both replicates displayed similar read distributions, gene targets and reproducible peaks (Figure 3C, D, Supplementary Figure S6). Strikingly, we found that PKM2 was crosslinked primarily to the coding sequence (CDS) of target transcripts encoding primarily cytosolically localized proteins (Figure 3C–E). This suggested that PKM2 is in close proximity to translating mRNAs, as expected for our results and a previous study which identified PKM as a ribosome-associated protein (10). Additionally, we found that purified GST-PKM1 co-sedimented with salt-washed ribosomes *in vitro*, again suggesting that PKM1 and PKM2 likely behave similarly in their capacity to interact with polysomes (Figure 3F). We were able to further verify the binding of GST-PKM1 to ribosomes using microscale thermophoresis (MST) (58,59), which has been used previously to characterize interactions between ribosomes and their binding proteins (60,61). When the thermophoretic mobility of fluorescein labeled GST-PKM1 was measured in increasing concentrations of ribosomes (Figure 3G), we were able to obtain a dose response curve (Figure 3H) that was used to estimate a dissociation constant (*K*_d_) of ≈ 0.9 ± 0.2 μM. Although we were able measure interactions at relatively high concentrations of ribosomes (1.5 μM at the highest MST concentration), we were unable to fully saturate PKM binding. In cell extracts, endogenous PKM co-sedimented with puromycin treated ribosomes, which are not bound to mRNA and nascent chains; however, this association was disrupted by high salt (Figure 3I). PKM also co-sedimented with ribosomes from cell extracts treated with cycloheximide regardless of salt concentration (Figure 3I). Since cycloheximide maintains the association between ribosomes, mRNA and nascent chains, our observations suggest that PKM may form additional contacts with either the mRNA or nascent polypeptide chain. In contrast, poly(A) binding protein (PABP) co-sedimented with ribosomes under all conditions (Figure 3I), consistent with previous findings (62). Since the binding of PKM to polysomes is partially RNase-sensitive (Figure 1G), it is possible that PKM recognizes rRNA, as suggested by previous work (10). While we observe PKM2 eCLIP peaks on sections of mature rRNA (Supplementary Figure S7A–C) they do not align with previously-identified PKM2 CLIP reads, and was not significantly enriched over the input (Supplementary Figure S7D). Nevertheless, it remains possible that the detection of PKM-rRNA association is highly dependent on the crosslinking procedure used.

Although our metagenome plot would suggest that PKM2 has no underlying sequence bias (Figure 3D) we found that it had well defined crosslinking sites to individual target mRNAs. For example, in the case of the *G3BP2* mRNA, we found PKM2 crosslinking to regions encoding peptides they are rich in glutamate and lysine (Figure 3J). We analyzed all PKM2 mRNA targets and found that binding to sequences encoding charged residues was a general feature of PKM2 crosslinking sites (Figure 3K, L). A close examination of the distribution of encoded amino acids revealed that the peak enrichment was slightly upstream of the eCLIP crosslink (Figure 3L, left of dotted line) suggesting that the recruitment of PKM to polysomes occurs after the synthesis of charged polypeptide chains. We plotted the enrichment of all amino acids proximal to the PKM2 eCLIP crosslinking site and found that only glutamate and lysine were significantly enriched over background (Figure 3M, Supplementary Figure S8) suggesting that these two amino acids predominantly contributed to the bias in charged residues.

### PKM promotes ribosome pausing and decay of substrate mRNAs

**Figure 4.**
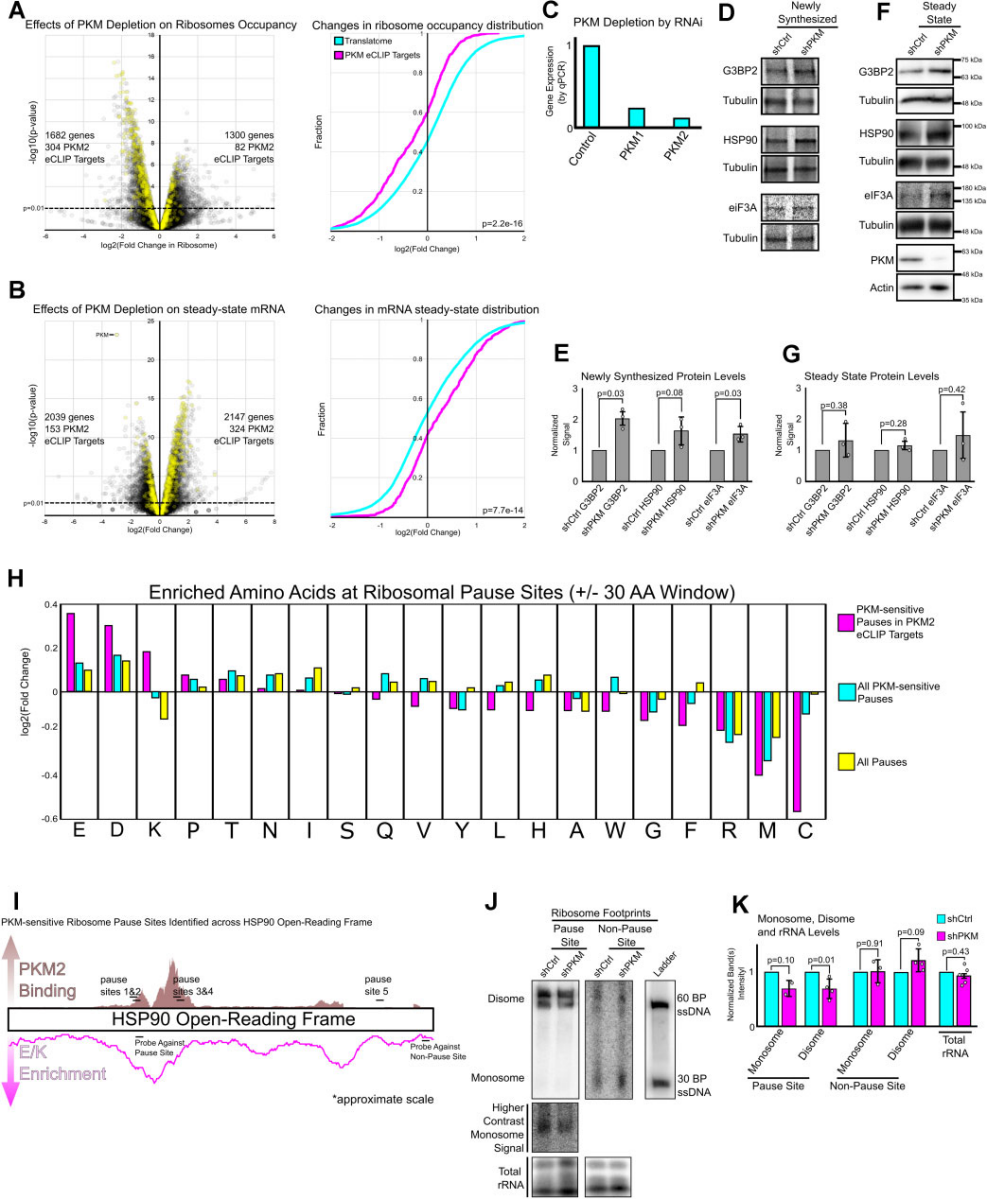
PKM inhibits the translation elongation of ribosomes on a subset of mRNAs. (A, B) PKM was depleted from HEK293T cells using lentiviral-delivered shRNAs. Lentiviral-delivered scrambled shRNA was used for control cells. Ribosome profiling was performed and the fold change in ribosome occupancy (A, dot plot) and steady-state RNA levels (B, dot plot) upon PKM depletion for each transcript was plotted. PKM2 eCLIP targets are highlighted in yellow. Cumulative distribution functions for the fold change in ribosome occupancy (**A**) and steady-state RNA levels (**B**) were plotted for PKM2 eCLIP targets (magenta) and the translatome (cyan; all detected transcripts). *P*-value for CDF (A, B) calculated from Kolmogorov-Smirnov test. (**C**) Depletion of mRNA levels of PKM (M1 & M2 isoforms) normalized to GAPDH (control) as measured by qPCR upon lentiviral-delivered shRNAs targeting PKM. (D, E) Newly synthesized proteins were labeled by feeding control or PKM-depleted U2OS cells with ^35^S-methionine/cysteine for 15 min, and G3BP2, HSP90AA1, eIF3A and α-tubulin were immunoprecipitated. The newly synthesized proteins were separated by SDS-PAGE, imaged by autoradiography (**D**), and levels were analyzed by densitometry analysis (**E**). Each bar represents the mean of at least three biological replicates plotted alongside standard error, as whiskers, and individual replicates, as dots. *P*-value calculated from paired Student's *t*-test. (F, G) Immunoblot of G3BP2, HSP90AA1, eIF3A and PKM in U2OS cells. Steady state levels quantified by densitometry analysis (**G**). Each bar represents the mean of at least three biological replicates plotted alongside standard error, as whiskers, and individual replicates, as dots. *P*-value calculated from paired Student's *t*-test. (**H**) The enrichment of all 20 amino acids within the vicinity of predicted ribosome pause sites (+/- 30 amino acid window). Yellow bars are all detected pause sites. Cyan bars are pause sites that are sensitive to PKM depletion. Magenta bars are pauses found within PKM2 eCLIP targets that are also PKM-sensitive. (**I**) The distribution of PKM2 eCLIP reads (magenta) across *HSP90AA1* open-reading frame and the enrichment of glutamic acid and lysine across the HSP90AA1 polypeptide (in a 30 amino acid moving average). Predicted ribosome pause sites are indicated by black bars and labelled. Northern blot probing sequences are indicated by black bars and labelled. (**J**) Northern blot of ribosome footprints at predicted *HSP90AA1* pause sites and non-pause sites in shCtrl-treated and shPKM mediated depleted cells. Monosome, disomes and high-molecular weight ribosomal species are indicated and labelled based off molecular weight ladder. Monosome signal was captured at higher exposure for increased visibility. Total rRNA was stained by ethidium bromide. (**K**) Densitometry analysis of footprint and rRNA signals as shown in (J). Note that due to the low levels of monosome signals, it was not always visible in all four experiments. We only quantified those experiments where the monosome signal was visible. Each bar represents the mean of at least 3 biological replicates plotted alongside standard error, as whiskers, and individual replicates, as dots. *P*-value calculated from paired Student's *t*-test.

Previously, it was found that PKM depletion resulted in a decrease in ribosomal occupancy for a subset of mRNAs (10). We depleted PKM in HEK293T cells by RNAi using lentiviral-delivered shRNAs and performed ribosome profiling sequencing (Figure 4A–C, Supplementary Figure S9). As expected, the peak fraction of reads fell in the 28–32 nucleotide range. Metagene plots indicated high coverage across the ORF alongside conventional trinucleotide periodicity and strong correlations were found between replicates (Supplementary Figure S9A–D). Overall, PKM depletion significantly affected the ribosome occupancy of mRNAs from almost 3000 genes (1682 decreased occupancy versus 1300 increased occupancy, *P*-value <0.01; Figure 4A, Supplementary Table S4). PKM2 eCLIP targets were disproportionally affected and tended to have decreased ribosome occupancy in PKM-depleted cells (Figure 4A, yellow dots). Additionally, PKM depletion significantly affected the steady-state level of mRNA from 4186 genes (2147 upregulated versus 2039 downregulated, *P*-value < 0.01) (Figure 4B). Again, we found that PKM2 eCLIP targets were disproportionally affected and tended to be stabilized by PKM depletion (Figure 4B, yellow dots).

Since ribosome profiling only provides a snapshot of translation, we were unable to differentiate whether high ribosome footprint counts on a given mRNA was due to an increase in translation initiation or elongational stalling (63). The former possibility would lead to an increase in protein production, while the latter would result in a decrease. In particular, there is growing evidence that sub-optimal elongation is coupled to mRNA decay (64) which may be the case for PKM eCLIP targets. This would explain the stabilization of these mRNAs upon PKM depletion (Figure 4B). To distinguish between these two possibilities, we measured the synthesis of proteins (G3BP2, HSP90AA1 and eIF3A) that are encoded by mRNAs that are both PKM eCLIP targets and whose ribosome occupancy is affected by PKM depletion. Newly synthesized proteins were labelled by a ^35^S-methionine/cysteine pulse, and isolated by immunoprecipitation. PKM depletion increased the synthesis of G3BP2, HSP90AA1 and eIF3A (Figure 4D, E). We also observed a general increase in the steady level of these proteins, although these were not statistically significant (Figure 4F, G). Overall, these observations are consistent with a model that PKM preferentially interferes with translational elongation on a subset of mRNAs.

Using our ribosome profiling dataset, we mapped putative pause sites in mRNAs present in all control and PKM-depleted replicates with high expression (RPKM > 1), with mRNAs from 605 genes meeting this cut-off. We then identified pause sites as regions with an unexpectedly higher preponderance of ribosome footprints than expected. In total, we mapped 5244 pause sites in mRNAs from 541 genes. Of these pause sites, 1298 were PKM-dependent (present in 440 mRNAs), with almost all (98.3%) having lowered ribosome read counts upon PKM depletion. Of the PKM-dependent sites, 500 were found across 131 PKM eCLIP target mRNAs and all had lower read counts after PKM depletion. We found that PKM-sensitive pauses in eCLIP-targets were present in regions coding for polypeptides enriched in glutamate and lysine (magenta bar, Figure 4H), similar to PKM crosslinking sites (Figure 3L). To validate this finding, we performed a northern blot and probed for a predicted ribosome pause site in the *HSP90AA1* mRNA (Figure 4I) in nuclease treated lysates–which should be protected from degradation by paused ribosomes. We found a decrease in signal for the monosome and disome fragments in PKM depleted cells (Figure 4J, K). In contrast, mRNA signal from probes against non-pause sites in the *HSP90AA1* mRNA was not affected by PKM depletion (Figure 4J, K).

### Nascent polypeptide chains are PARylated, which is required for PKM-polysome association

**Figure 5.**
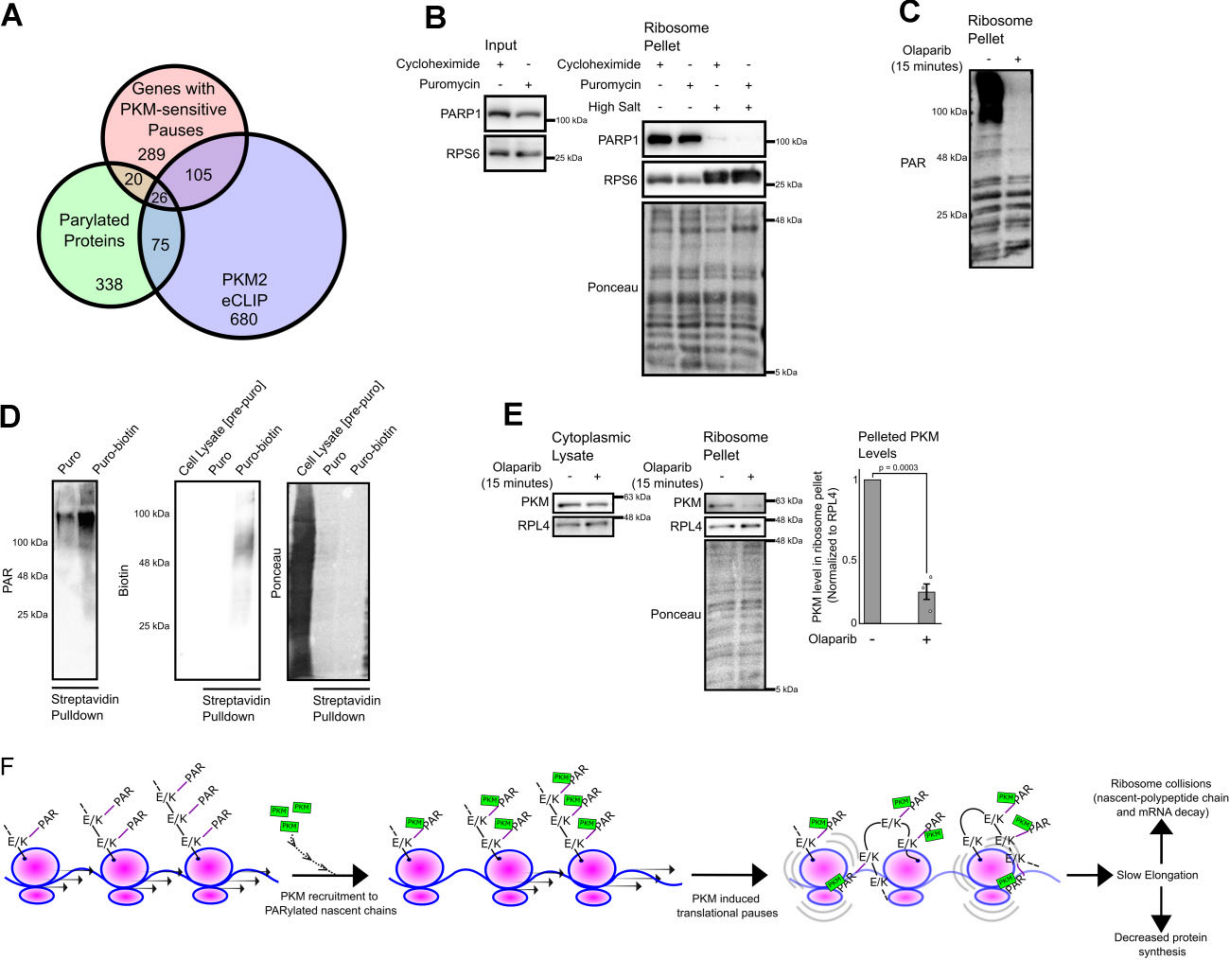
PKM associates with polysomes that contain PARylated nascent chains. (**A**) Overlap between PKM2 eCLIP targets, mRNAs containing PKM-sensitive ribosomal pauses and PARylated proteins (from Martello *et al.* 2016). (**B**) U2OS cells were treated with either 200 μM Puromycin (200 μM) or Cycloheximide (100 μg/ml) for 10 min and then lysed and incubated in isotonic (125 mM KCl) or a high salt (500 mM KCl) buffer. Samples were either directly analyzed (‘Input’) or polysomes were isolated by centrifugation through a sucrose cushion and the pellets were analyzed by immunoblot for PARP-1 and RPS6 levels. Ponceau was used to monitor total protein. (**C**) U2OS cells were treated with PARP-1 inhibitor Olaparib for 25 min. Polysomes were isolated by sucrose cushion sedimentation and pellets were analyzed by immunoblot for poly-ADP ribosylated (PAR) proteins. (**D**) Polysome pellets were incubated with either puromycin or biotin-tagged puromycin. Streptavidin pulldown was performed to isolate biotin-tagged puromycinylated nascent polypeptides. Pulldown analyzed by immunoblot and probed for poly-ADP ribosylated nascent chains (PAR), biotin and total proteins monitored by Ponceau. (**E**) U2OS cells treated with PARP-1 inhibitor Olaparib for 25 min. Samples were either directly analyzed (‘Input’) or polysomes were isolated by sucrose cushion sedimentation and pellets were analyzed by immunoblot for PKM, RPL4 and total proteins monitored by Ponceau. PKM signal was quantified relative to RPL4 levels in ribosome pellets. Each bar represents the mean of at least three biological replicates plotted alongside standard error, as whiskers, and individual replicates, as dots. *P*-value calculated from paired Student's *t*-test. (**F**) Model depicting the recruitment of PKM to polysomes and their subsequent fate.

Lysine-rich nascent chains encoded by poly(A)-stretches are known to induce translational stalling (65,66), however, the impact of glutamate-rich nascent chains on translation is unclear. Despite this, four studies have identified glutamate to be enriched near endogenous stall sites (67–70). Given the disparate chemical characteristics of both lysine and glutamate, we suspected that a transient modification on the nascent polypeptide rather than the underlying amino acid sequence recruited PKM to the ribosome. Both positive and negatively charged amino acids are known to be the target of a cellular modification known as poly-ADP ribosylation (PARylation) (71). Notably, a previous study found that PKM can directly bind to PARylated polypeptides (35). Furthermore, PKM was identified in a mass spectrometry screen for PAR-binding proteins (72). Additionally, many PKM eCLIP targets and mRNAs whose pauses are PKM sensitive are significantly enriched for PARylated proteins (Figure 5A, respective *P*-values for enrichment: 4.00 × 10^−43^, 3.83 × 10^−18^). Incidentally, we found the major PARylation polymerase (PARP-1) in our polysome mass spectrometry screen, and further validated this interaction by immunoblot (Supplementary Table S1, Figure 5B). Probing for PARylated proteins in our polysome pellets reveals a slow migrating smear near the top of the gel (Figure 5C). Additionally, we also identified banding pattern akin to ribosomal proteins (Figure 5C)–consistent with a study indicating that ribosomal subunits are PARylated (73). Remarkably, pre-treating cells with the PARP inhibitor, olaparib, for 15 min eliminates the slow migrating species suggesting that these PARylated substrates have a short half-life, in contrast to the putative PARylated ribosomal bands (Figure 5C). We hypothesized that the slow migrating species consisted of PARylated nascent polypeptides whose apparent molecular weight is drastically altered by PARylation. To test this hypothesis, we tagged nascent polypeptides using a biotinylated-puromycin analogue and isolated them via a streptavidin pulldown. We were able to recover a slow migrating PAR-enriched species in our pulldown (Figure 5D, PAR blot), which was of higher apparent molecular weight than total nascent polypeptides as detected by probing for biotin (Figure 5D). In contrast, we did not detect the numerous low molecular weight PARylated bands in the streptavidin pulldown, concomitant with a loss in ribosomal proteins as detected by ponceau stain (Figure 5D). Finally, we found that olaparib treatment disrupted the co-sedimentation of PKM with polysomes, suggesting that PARylation may be required for PKM binding (Figure 5E).

## DISCUSSION

Here, we present evidence that PKM associates with cytosolic polysomes to promote translational pausing in the presence of glucose and pyruvate. We propose a model (Figure 5F) where under normal conditions, PKM binds to PARylated nascent chains via its ADP-binding site. At this moment, we are unable to distinguish whether PKM stalls translation in *cis* (i.e. its bound ribosome) and/or in trans (i.e. neighbouring ribosomes). Given the flexibility and length of nascent chains and PAR-chains, both mechanisms may be happening simultaneously. The recruitment of PKM to nascent chains allows it to stall elongation, likely by associating with the A-site of nearby ribosomes as indicated by Simsek et al (10). This model is reminiscent of how the signal recognition particle inhibits elongation by occluding the A site and thus preventing the entry of tRNAs (74). In agreement with this model, it has been recently found that the *E. coli* homologue of PKM interacts with the ribosome A site, suggesting that this may be an evolutionary conserved mechanism (75). Then upon glucose/pyruvate starvation, increased levels of ADP compete with PAR for PKM-association and relieve this elongation inhibition. This model of ADP regulation is in agreement with previous observations that ADP and PAR bind to the same pocket on PKM (35), and this is likely due to fact that PAR chains are in part composed of ADP-moieties.

Our data indicates that this PKM-dependent regulation is restricted to cytosolic polysomes. Notably, ER-associated polysomes have their nascent chains translocated into the ER lumen and thus would not be accessible to either PARP-1 or PKM. This is in agreement with our observations that PKM does not associate with ER-associated polysomes (Figure 1G). While our model suggests that nascent chains are co-translationally PARylated, we are unable to rule out an indirect effect given that an increase in PAR polymers can affect cellular metabolism via a decrease in NAD+ levels (76). While our PKM2 eCLIP targets do not completely overlap with the set of PARylated proteins from Martello et al., we suspect that this may reflect the difficulties of detecting PARylation rather than an absence of PARylation, especially those occurring on nascent polypeptides. It must be noted that a previous group had reported ADP-ribosylation activity associated with cellular ribosomes that could be partially reduced by inhibiting protein synthesis (77).

We suspect that elongation rates may affect protein folding and that both of these processes require active feedback from glycolysis. Under normal conditions, PARylation may allow poorly folded domains to remain soluble as they exit the ribosome and may induce translational stalling through PKM to further promote proper folding of the nascent polypeptides. This is analogous to how protein glycosylation may help to fold proteins in the lumen of the ER (78). Poorly folded nascent polypeptides would eventually promote PKM-dependent ribosome collisions and thus activate cleavage of the translated mRNAs and the decay of partially synthesized unfolded nascent chains. Notably, PAR accumulates in stress granules (79), large cytoplasmic biomolecular condensates of mRNA and RNA-binding proteins that form in response to cellular stress (80). However, under starvation conditions when ADP levels rise, we speculate that this process is inactivated to allow the cell to conserve energy by preventing the decay of energetically costly mRNAs and nascent polypeptides. Consistent with this model is the observation that nearly a quarter of newly synthesized cytosolic proteins are rapidly degraded (81). Furthermore, it has been shown that PKM depletion enhances sensitivity to proteasome inhibition in mammalian cells by mediating the formation of the CHIP-HSP70-BAG3 complex with ubiquitinated proteins (82). Since BAG3 has been shown to associate with polypeptides as they exit the ribosome, this finding is in agreement with our model that PKM is required to eliminate poorly folded nascent proteins.

Overall, PKM depletion induces a variety of cellular phenotypes that include dysregulated carbohydrate metabolism, mitotic defects, DNA damage defects and mRNA translation (10,30,32,34,35,83–87), suggesting that it may have many roles in several distinct cellular processes. It is possible that our newly characterized role of PKM may explain how these processes are linked. Among the transcripts whose ribosome occupancy are PKM-sensitive and/or interact with PKM by eCLIP, we find an enrichment for proteins involved in DNA repair, DNA replication, and mitotic regulation (Supplementary Table S3 and S4). Furthermore, the PKM-ribosome interaction is lost during mitosis (Supplementary Figure S5D), at the exact same time when ribosome-associated PARylation drops (77)–suggesting that elongation of mitotic-encoding transcripts may be enhanced during cell division. Additionally, it must be noted that PARylation has a close relationship with DNA damage as it is an important transient modification which acts to recruit DNA repair factors to damaged DNA regions. It has been shown that DNA-damage can reduce protein synthesis (73) and trigger stress granule assembly, a hallmark of reduced mRNA translation (88,89). Interestingly, DNA damage has been shown to recruit PKM to the nucleus in a PAR-dependent manner (35). Whether PKM and/or PAR mediate how DNA damage triggers the repression of mRNA translation remains to be elucidated.

## DATA AVAILABILITY

The proteomic data can be accessed through ProteomeXchange (PXD038978) or Massive (MSV000090938). The PKM eCLIP data are available at https://s3-us-west-1.amazonaws.com/sauron-yeo/20170112_PKM2_CLIP/hub.txt and can be accessed through the UCSC Genome Browser (https://genome.ucsc.edu/cgi-bin/hgHubConnect#unlistedHubs). The ribosome profile sequencing data can be access through the GEO database (GSE202881).

## Supplementary Material

gkad440_Supplemental_Files

## Notes

Present address: Lena Ilan, Syntezza Bioscience, Jerusalem, Israel.

Present address: Ines Rabano, Illumina, Cambridge, United Kingdom.
