# Supplementary material for: Pyruvate Kinase M (PKM) binds ribosomes in a poly-ADP ribosylation dependent manner to induce translational stalling
Source: Nucleic Acids Res. 2023 May 24;51(12):6461–78. doi: 10.1093/nar/gkad440 (PMC10325899; doi:10.1093/nar/gkad440)
Supplement: gkad440_Supplemental_Files [file gkad440_supplemental_files.zip › Kejiou et al. Supplementary Figures, Captions and Legends.pdf]

Supplemental Figure 1. Distribution of peptides from glycolytic proteins isolated by oligo-dT affinity chromatography.

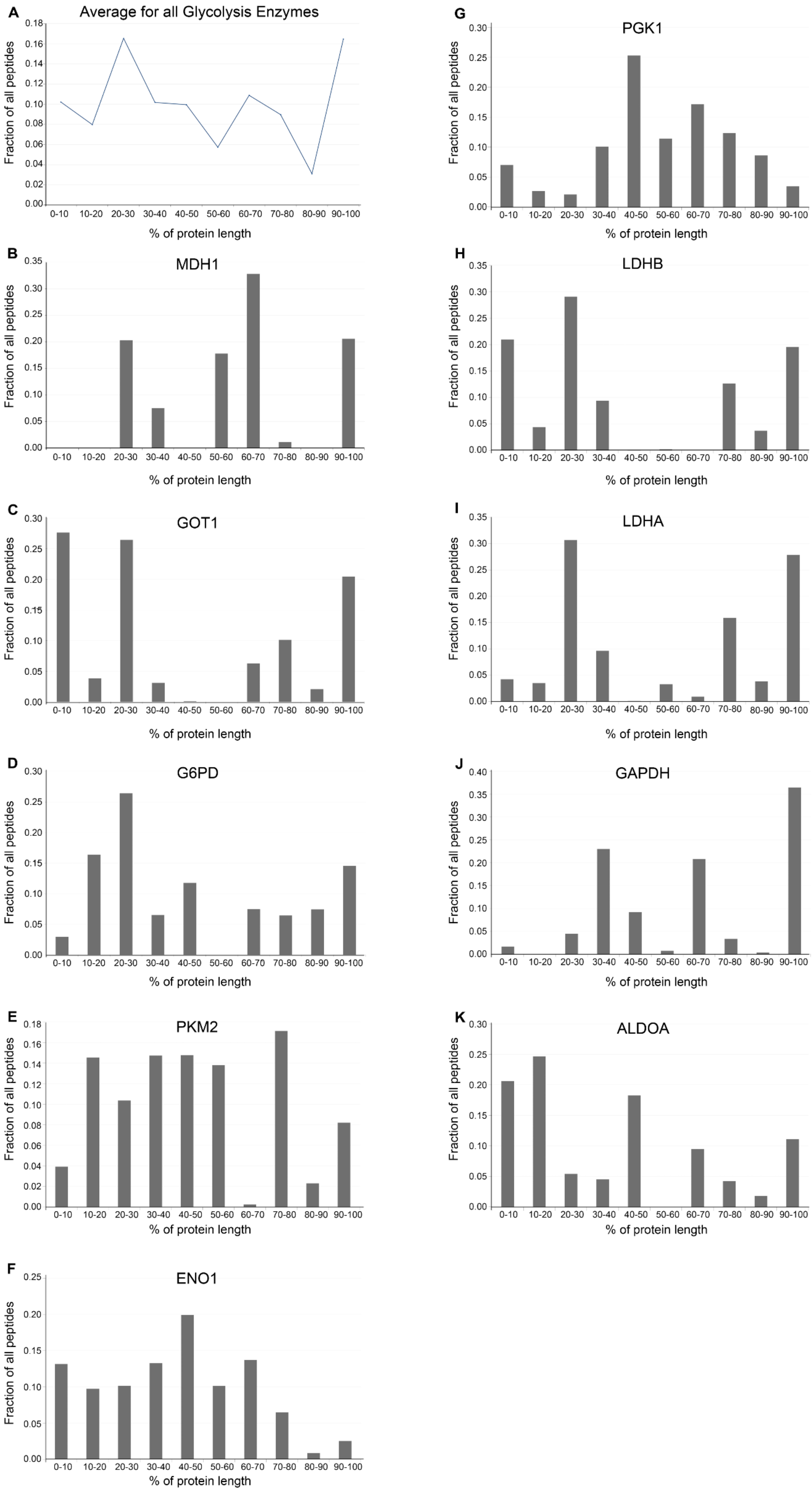

Supplemental Figure 2. The distribution of many ribosome-associated proteins differs significantly between the ER and cytosol.

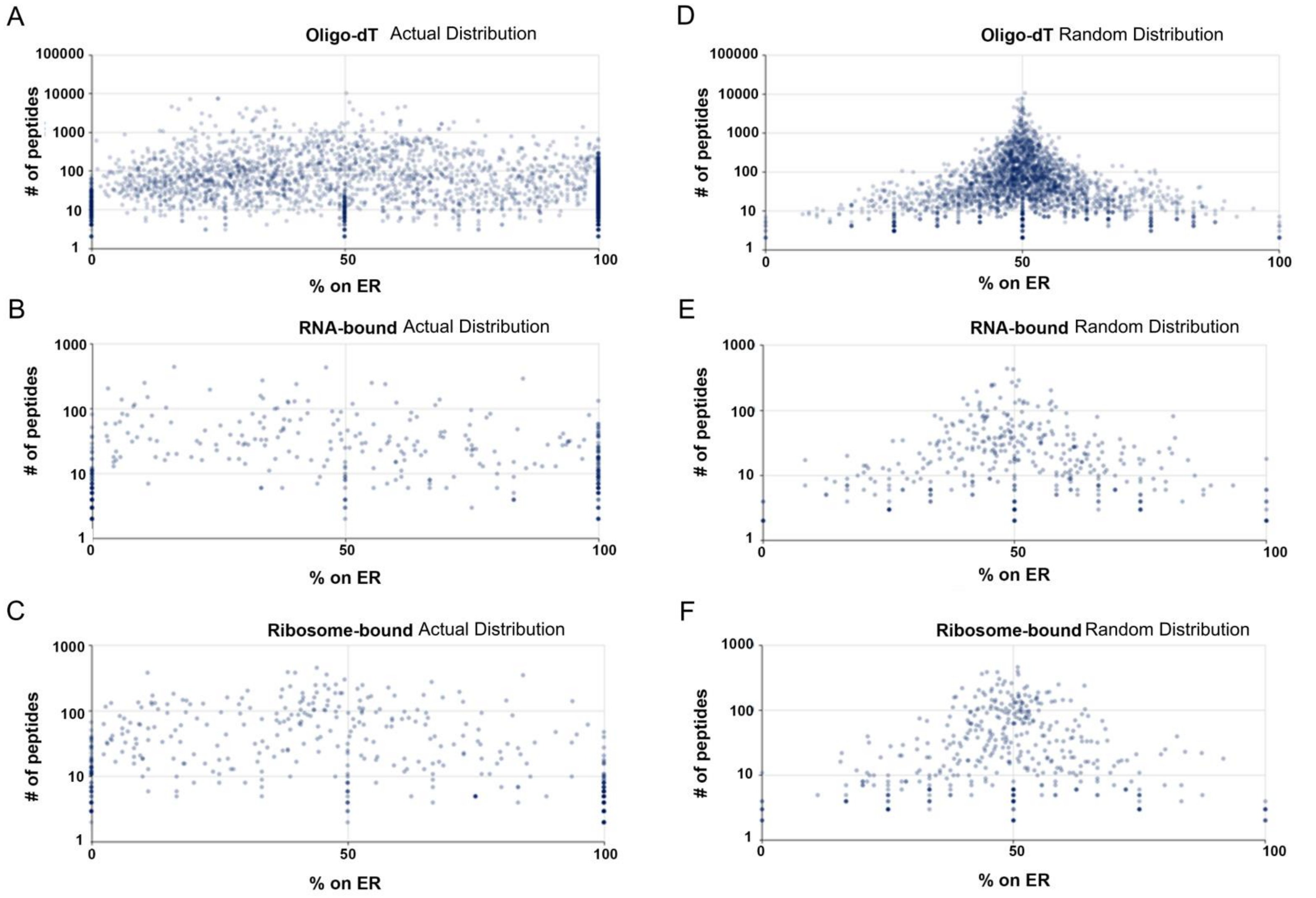

Supplemental Figure 3. Ribosomal proteins were equally sampled between the ER and cytosol.

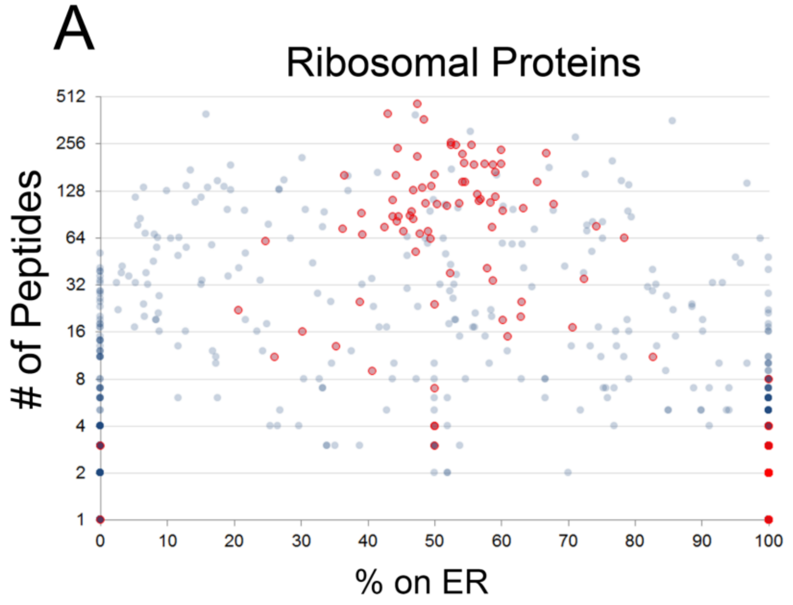

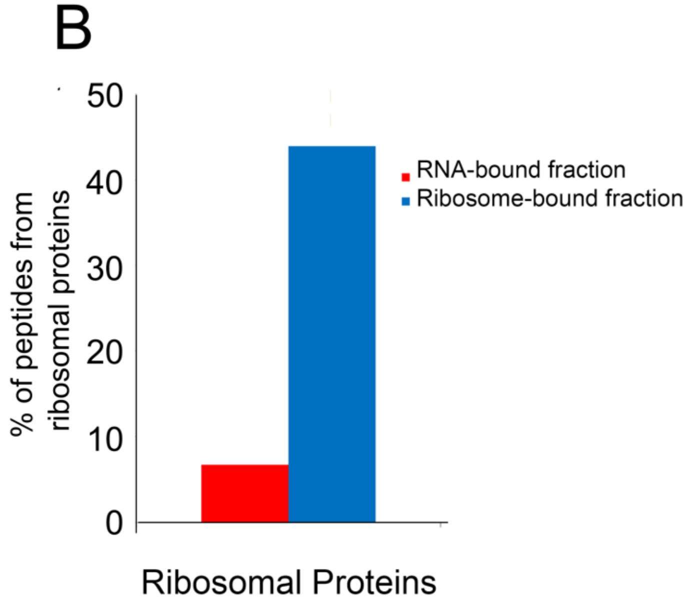

Supplemental Figure 4. ER/Cytosolic Distribution of RNA and ribosome-associated proteins

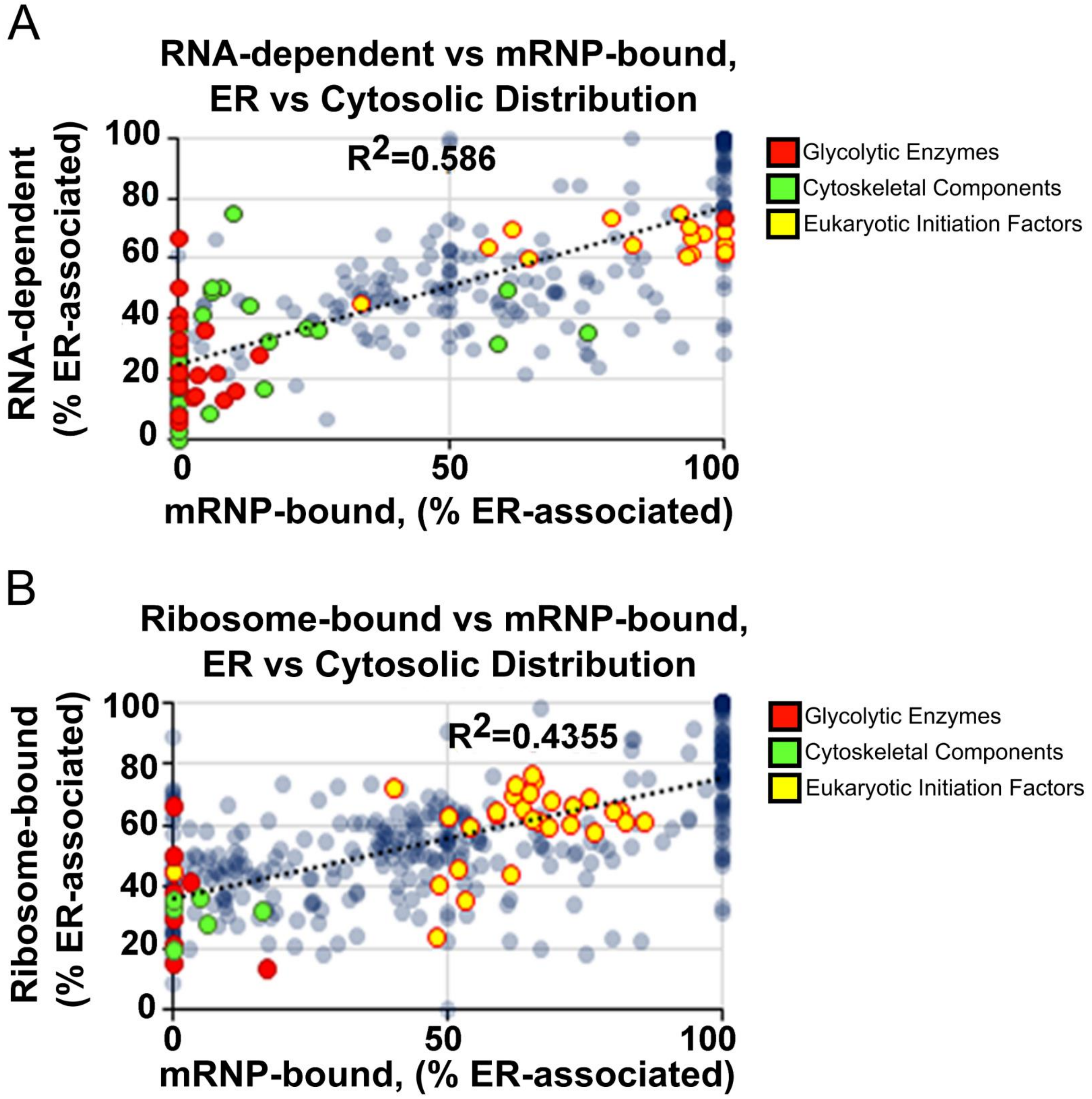

Supplemental Figure 5. PKM differentially associates with ribosomes depending on drug or metabolic pre-treatment, and mitotic status

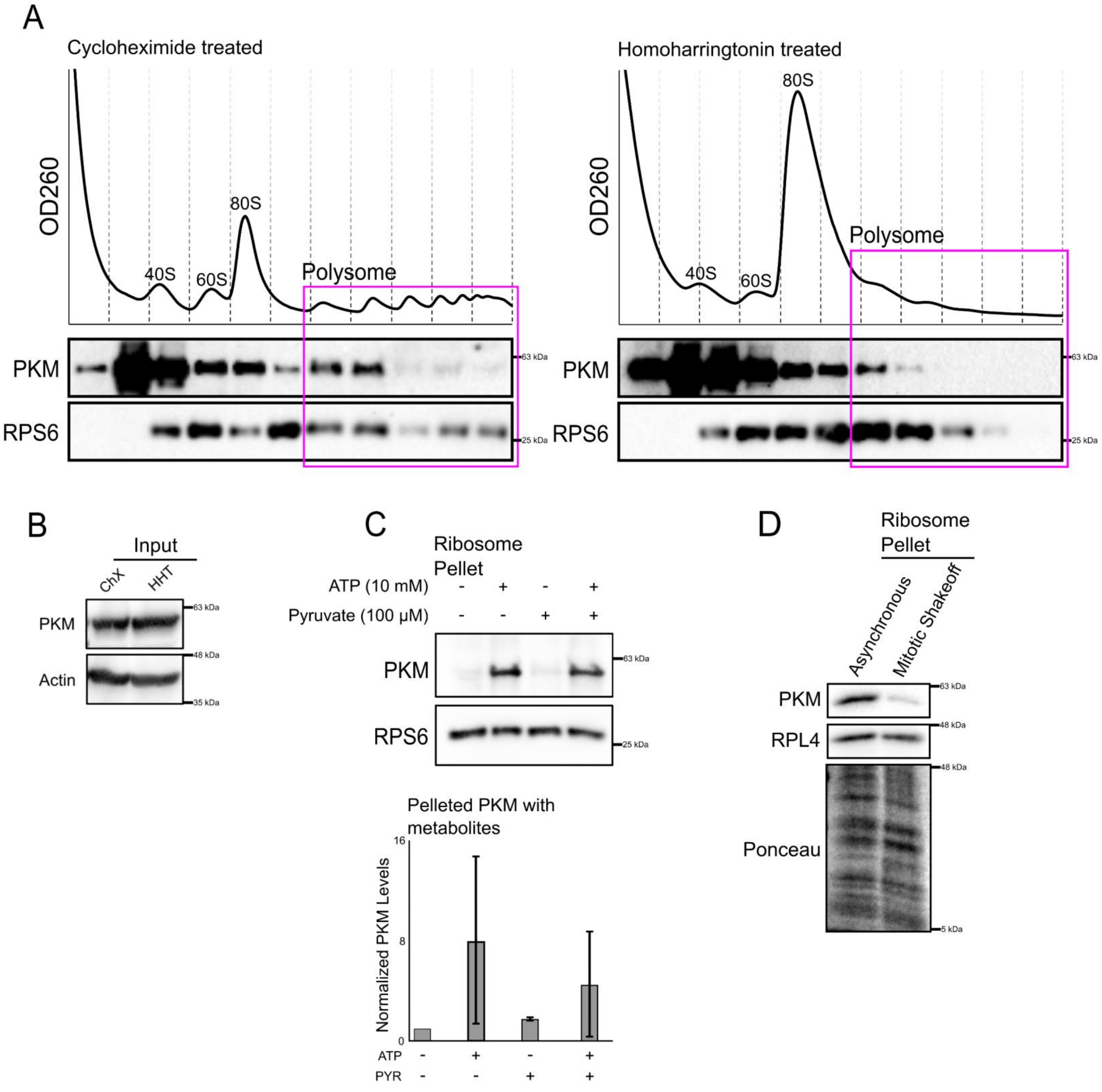

Supplemental Figure 6. PKM2 eCLIP Quality Control

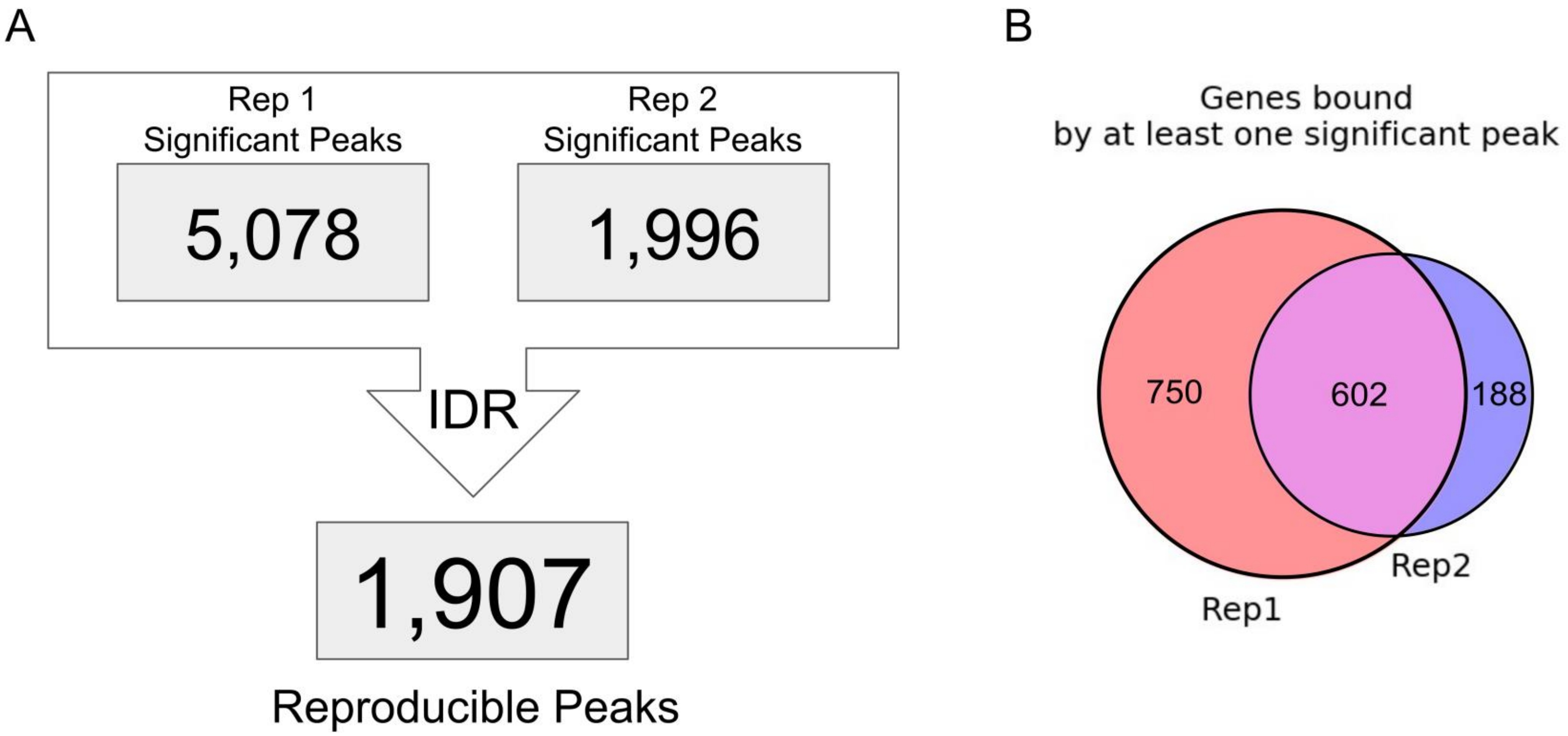

Supplemental Figure 7. PKM2 eCLIP peaks mapping to rRNA.

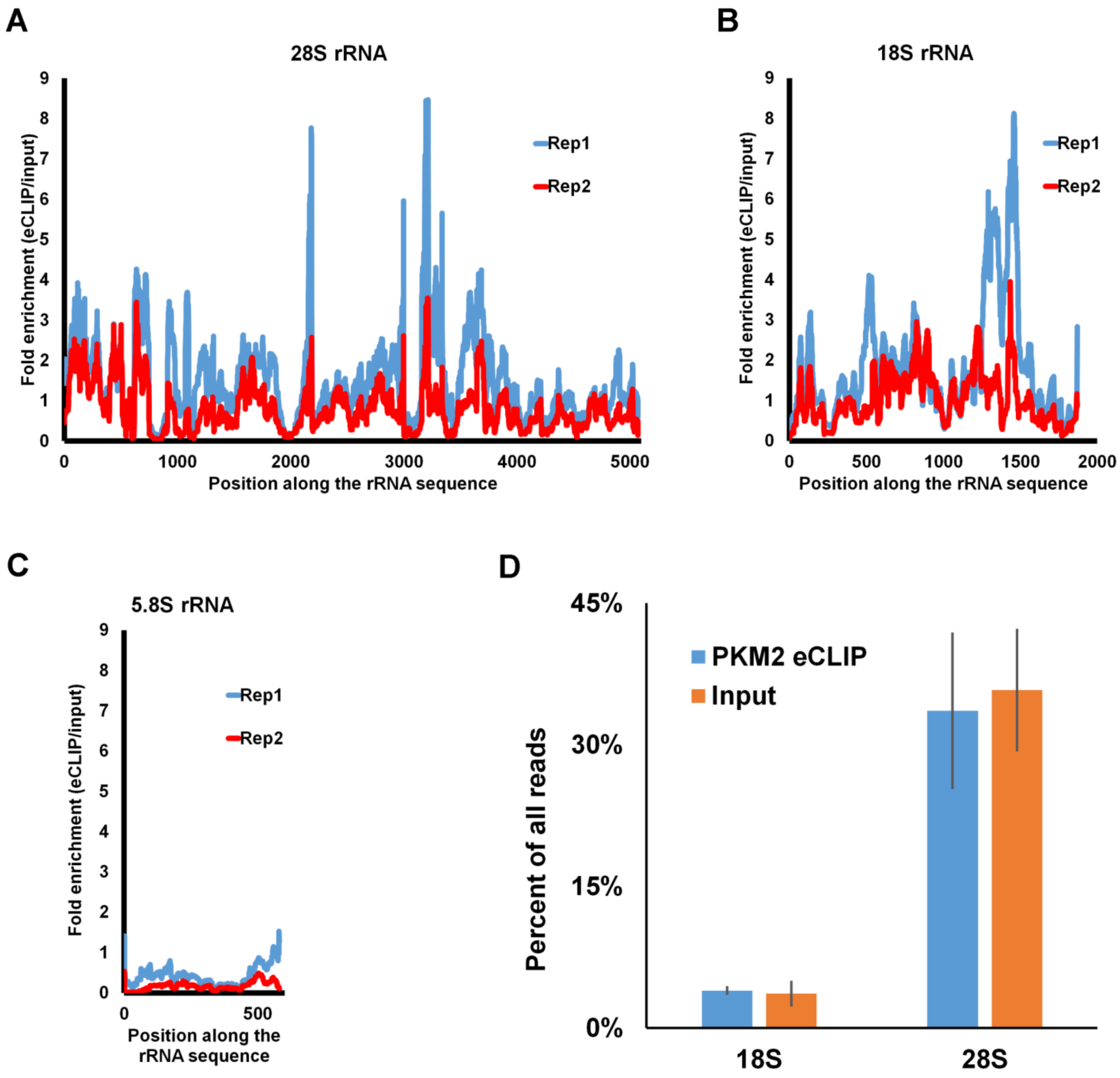

Supplemental Figure 8. Frequency of amino acids near PKM eCLIP crosslinking site

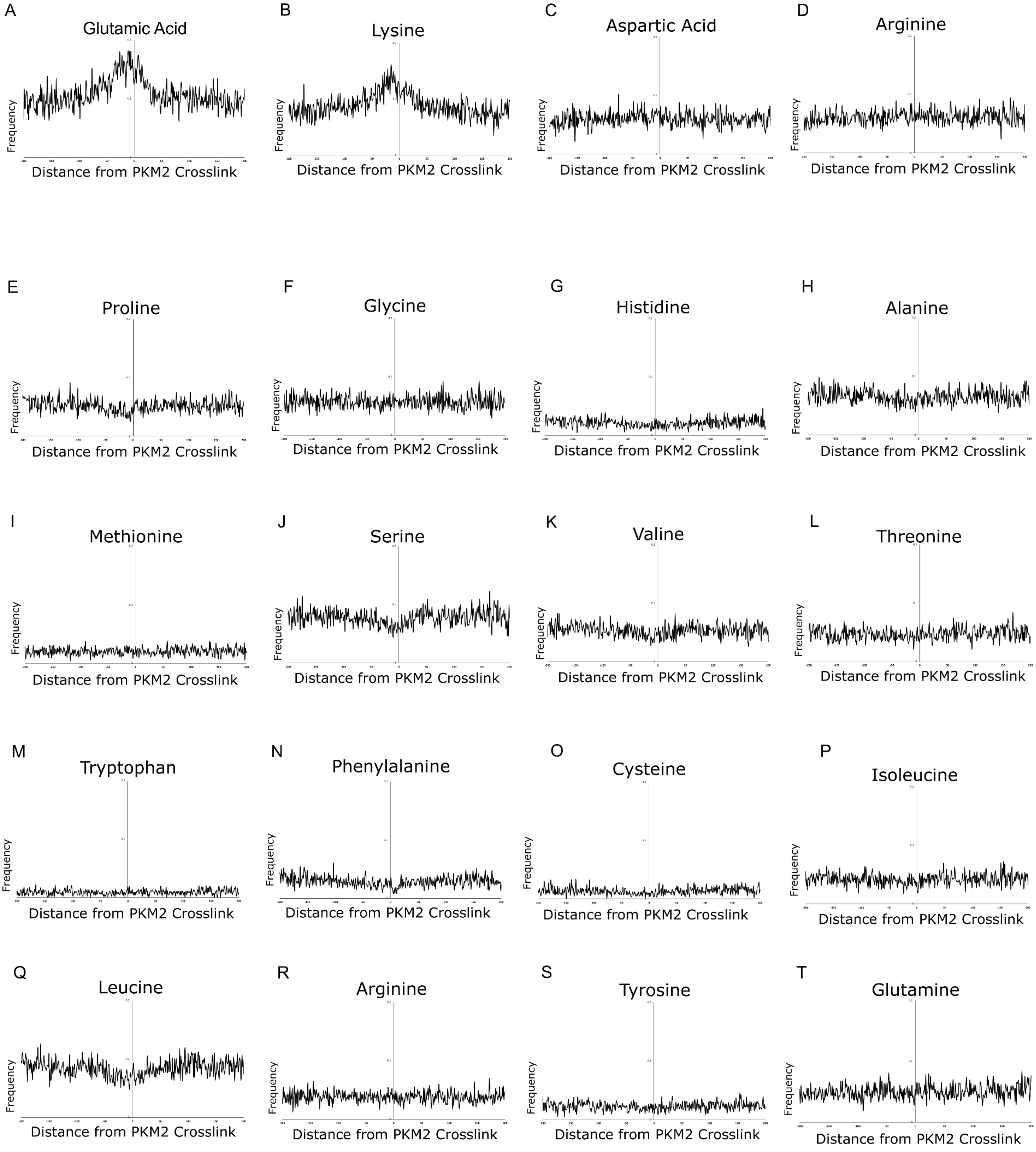

Supplemental Figure 9. Ribosome profiling quality control

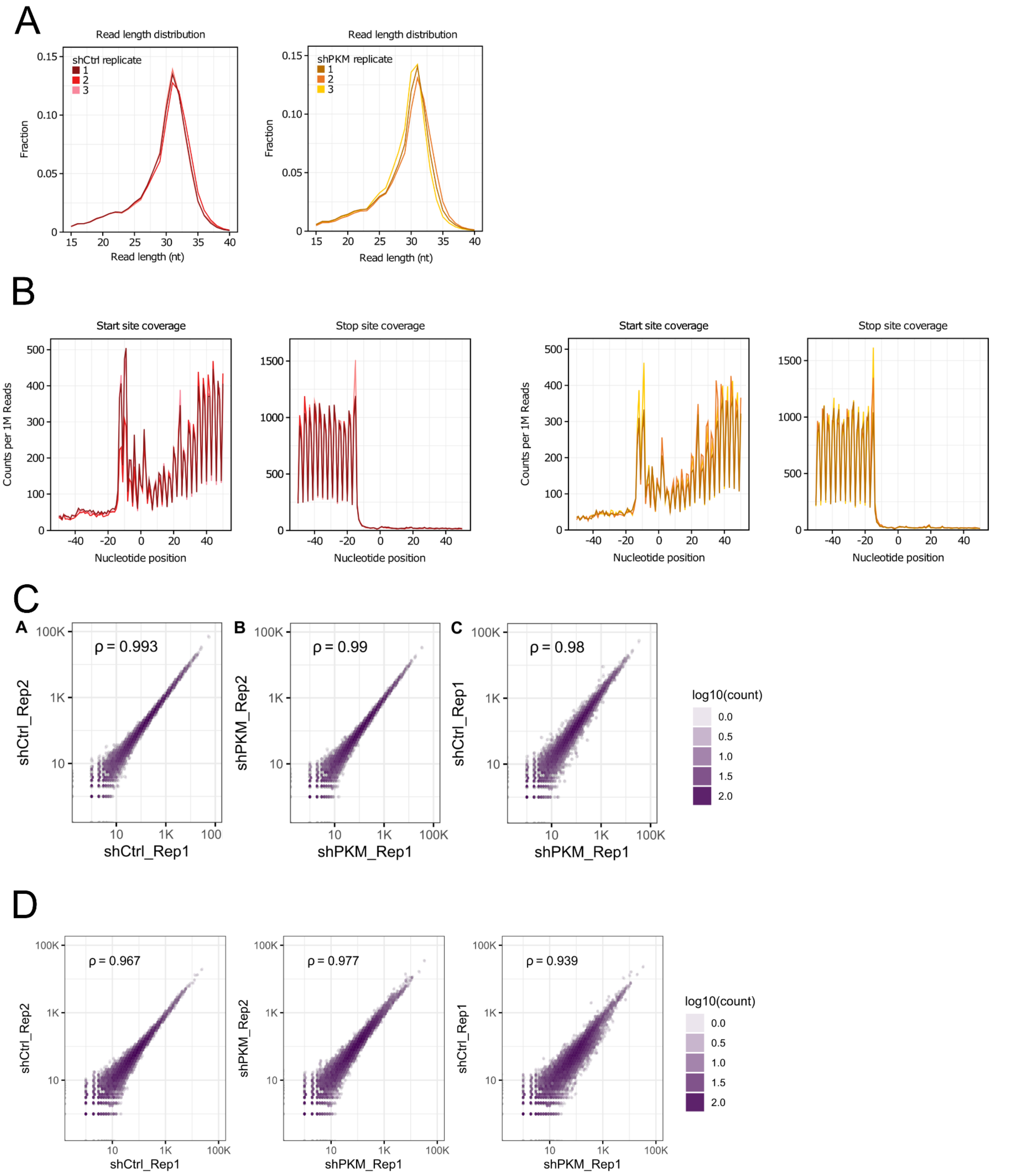

Supplemental Figure 1. Distribution of peptides from glycolytic proteins isolated by oligo-dT affinity chromatography.

For all the listed proteins, the number of spectral counts for peptides along the length of the protein were compiled and binned. The distribution of the fraction of total peptides (*y-axis*) along the protein length for each bin (from N- to C-terminus; *x-axis*) were plotted.

A) The overall average for all glycolysis enzymes. Note the large fraction of peptides that map back to the C-termini (bin 90-100%).

B-K) The distribution for individual genes. Note that with the exception of ENO1 and PGK1, a significant of peptides isolated in the oligo-dT affinity purifications map back to the C-termini (bin 90-100%).

Supplemental Figure 2. The distribution of many ribosome-associated proteins differs significantly between the ER and cytosol.

A-C) For each protein present in both crude ribosome and oligo-dT purifications in our list of mRNA/Ribosome-associated proteins (see Supplemental Table 1), the total number of peptides (*y-axis*) identified by mass spectrometry was plotted against the percentage of peptides found in the ER [100% x peptides in ER/(peptides in ER + peptides in cytoplasm); '% on ER' - *x-axis*]. Note that data in (A-C) is identical to that of Figure 1J-L.

D-F) The same as (A-C) except that each peptide was randomly reassigned to either the ER or cytosolic fraction and the '% on ER' was recalculated.

Supplemental Figure 3. Ribosomal proteins were equally sampled between the ER and cytosol.

A) For each protein in our list of ribosome-associated proteins (see Supplemental Table 1), the total number of peptides (*y-axis*) identified by mass spectrometry was plotted against the percentage of peptides found in the ER [100% x peptides in ER/(peptides in ER + peptides in cytoplasm); '% on ER' - *x-axis*] for the ribosome-bound fractions. This data is identical to that of Figure 1K. Note that the ribosomal proteins,

which are labeled in red, are the most abundant proteins and are equally sampled between the ER and cytosol.

B) The normalized number of ribosomal peptides in the RNA-dependent fraction versus the ribosome-bound fraction. Overall ribosomal proteins made up approximately 10% of the peptides in the RNA-dependent fractions and 44% of the peptides from the ribosome-bound fractions.

Supplemental Figure 4. ER/Cytosolic Distribution of RNA and ribosome-associated proteins

Correlation between the percentage of peptides found in the ER/total in the RNA-dependent fraction of the crude ribosome preparation and in the oligo dT-associated proteins/mRNP-bound (A) and in the ribosomal-bound fraction of the crude ribosome preparation and in the oligo dT-associated/mRNP bound proteins (B). The data is identical to that of Figure 1J-L but replotted to compare the various fractions. Classes of proteins that are enriched in either the ER or the cytoplasm are highlighted as in Figure 1J-L – carbohydrate metabolic proteins (red), eIFs (yellow), and cytoskeletal-associated proteins (green).

Supplemental Figure 5. PKM differentially associates with ribosomes depending on drug or metabolic pre-treatment, and mitotic status

A) U2OS cells were treated with either 100 µg/mL cycloheximide (ChX) for 10 minutes to stabilize polysomes or 5 µM homoharringtonin (HHT) for 30 minutes to destabilize them. Cell lysates were sedimented through a 10-50% linear sucrose gradient at 36,000 RPM for 2 hours. Nucleic acids were monitored by OD260, and fractions were immunoblotted for pan-PKM (both PKM1 and 2), and RPS6.

B) Total cell lysate from U2OS pre-treated with either ChX or HHT as described in A) and probed for PKM and Actin by immunoblot.

C) U2OS lysate were spiked with a combination of either 100 µM Pyruvate, 10 mM ATP or both. Ribosomes were sedimented and probed for pan-PKM and ribosomes (RPS6). PKM signal was quantified relative to RPS6 levels in ribosome pellets. Each bar represents the mean of at least 3 biological replicates plotted alongside standard deviation, as whiskers.

D) Normal asynchronous, or mitotically-arrested (double thymidine block + nocodazole-treatment) U2OS cells were lysed, and crude ribosomes were sedimented. Sedimented fractions were probed for PKM and RPL4. Total proteins were also monitored by Ponceau stain.

Supplemental Figure 6. PKM2 eCLIP Quality Control

A) Significant peaks (defined by: -log10 p-value >= 3, log2 fold enrichment >= 3) from each replicate were merged using irreproducible discovery rate (IDR), yielding 1,907 reproducible peaks (consistency ratio: 5.37;rescue ratio: 2.40).

B) Venn diagram showing the overlap between genes with at least one significant peak in each replicate.

Supplemental Figure 7. PKM2 eCLIP peaks mapping to rRNA.

A-C) PKM2 eCLIP reads were compared to size-matched input reads using RNA repeat-family centric mapping (Van Nostrand et al., manuscript in prep.). The densities of eCLIP/input reads (*y-axis*) was plotted along the length (*x-axis*) of the 28S, 18S and 5.8S rRNA genes.

D) The fraction of PKM2 eCLIP and input reads vs total reads were plotted. Each bar represents the average and standard deviation of the two replicates.

Supplemental Figure 8. Frequency of amino acids near PKM eCLIP crosslinking site

A-T) The frequency of all amino acids relative to PKM eCLIP crosslinking site (denoted in center as position “0”).

Supplemental Figure 9. **Ribosome profiling quality control**

A) Read length distribution for ribosome profiling reads of shCtrl and shPKM replicates.

B) Metagene coverage for ribosome profiling reads for shCtrl and shPKM replicates.

C-D) Correlations of ribosome occupancy (C) and mRNA steady-state levels (D) between shCtrl and shPKM replicates.

Supplemental Table 1. Mass Spectrometry of mRNA/Ribosome-bound proteins

The list of 496 mRNA/Ribosome-bound proteins. Proteins must have peptides that appear in 1) at least two separate RNA-dependent or Ribosome-bound experiments (out of a total of five); and 2) in both mRNP-bound (oligo-dT affinity chromatography) experiments and have at least two-fold more peptides in the oligo-dT affinity chromatography precipitates than in the mock bead precipitates in both experiments. Each spectral count column represents the total counts for all experiments. Each percent ER column represents the average of at least 2 experiments. The percent ER is calculated by the fractional representation of spectral peptide counts for a given protein in the ER for each fraction (RNA-dependent; Ribosome-bound; mRNP-bound, see methods) divided by the fractional representation of spectral counts for the same protein in the ER and cytosol combined for that same experiment.

Supplemental Table 2. RBPome Comparisons

The presence of our 496 high confidence polysome interactors in previous mass spectrometry analyses of RNA and ribosome-interactomes

Supplemental Table 3. PKM2 eCLIP Peaks and Read Statistics

Supplemental Table 4. Ribosome Occupancy & mRNA level foldchange shCtrl versus shPKM
